# Mapping the value of commercial fishing and potential costs of offshore wind energy on the U.S: West Coast: Towards an assessment of resource use tradeoffs

**DOI:** 10.1371/journal.pone.0315319

**Published:** 2025-03-06

**Authors:** Blake E. Feist, Robert Griffin, Jameal F. Samhouri, Leena Riekkola, Andrew O. Shelton, Y. Allen Chen, Kayleigh Somers, Kelly Andrews, Owen R. Liu, Jennifer Ise

**Affiliations:** 1 Conservation Biology Division, Northwest Fisheries Science Center, National Marine Fisheries Service, National Oceanic and Atmospheric Administration, Seattle, Washington, United States of America; 2 Office of Research and Development, Environmental Protection Agency, Narragansett, Rhode Island, United States of America; 3 Natural Capital Project, Stanford University, Stanford, California, United States of America; 4 NRC Research Associateship Program, Conservation Biology Division, Northwest Fisheries Science Center, National Marine Fisheries Service, National Oceanic and Atmospheric Administration, Seattle, Washington, United States of America; 5 Fishery Resource Analysis and Monitoring Division, Northwest Fisheries Science Center, National Marine Fisheries Service, National Oceanic and Atmospheric Administration, Seattle, Washington, United States of America; 6 Ocean Associates Inc., under contract to the Northwest Fisheries Science Center, National Marine Fisheries Service, National Oceanic and Atmospheric Administration, Seattle, Washington, United States of Aerica; 7 West Coast Regional Office, National Marine Fisheries Service, National Oceanic and Atmospheric Administration, Long Beach, California, United States of America; MARE – Marine and Environmental Sciences Centre, PORTUGAL

## Abstract

The West Coast of the U.S. has a vast offshore wind energy (OWE) electricity generation potential with value on the order of billions of USD, and pressure is mounting to develop large OWE projects. However, this seascape has numerous existing resource extraction uses, including a multi-billion dollar commercial fishing industry, which create the potential for conflict. To date, spatially explicit comparisons of OWE and commercial fisheries value have not been done, but are essential for marine spatial planning and for investigating the tradeoffs of OWE development on existing marine uses. In this analysis, we generate maps of OWE levelized cost of energy and of total economic activity generated by the top eight commercial fishing targets that account for the vast majority (~84%) of landed revenue off the U.S. West Coast. We quantify spatial overlap between these two ocean uses and use multiobjective optimization to develop tradeoff frontiers to investigate implications for both sectors from established state goals or mandates for OWE power generation capacity. There are clear differences in the exposure of each fishery in their traditional fishing grounds as a function of differing OWE capacity goals and outcomes vary depending on whether OWE development goals are achieved at a state-by-state level or a region-wide level. Responsible siting of OWE projects includes careful consideration of existing commercial fishing activities, and responsible transition to renewable energies on the West Coast and elsewhere accounts for the socio-economic consequences of the total economic activity associated with each fishery.

## Introduction

Climate change is arguably the greatest anthropogenic environmental challenge facing humanity, and is an existential threat affecting virtually all Earth’s ecosystems and associated services [1]. The primary causative agent of this change is anthropogenic greenhouse gas loading in the form of carbon dioxide, methane and nitrous oxide. In 2022 approximately 37 Gt of CO_2_ was released by human activities [2], and current atmospheric CO_2_ concentrations are the highest they have been in 14 million years [3]. Combustion of fossil fuels for electric power generation is the greatest single source of CO_2_ emissions [1]; expedient conversion of the existing power grid to renewable energy is imperative if we seek to reduce the human carbon footprint and maintain a planet with conditions conducive to human habitability.

While the carbon footprint of renewable energy is much smaller compared with conventional fossil fuel based power sources, the land use footprint (land use area per MW produced) of renewable energy is generally larger [4–6]: the footprint of land (onshore) based solar and wind energy projects are three to 20 times larger than conventional fossil fuel based power sources [7–9]. Although the vast majority of existing renewable energy projects have been onshore [10], where there is increasing conflict with existing uses such as agriculture and urbanization [11], there is considerable impetus to aggressively develop offshore renewable energy projects, especially harvesting wind power [10,12,13]. The ocean will play a critical role in achieving the goal of dramatically decreasing carbon dioxide emissions, given it accounts for 71% of the Earth’s surface area and it has vast areas useful for a variety of renewable energy technologies ranging from wave to geothermal to wind [14]. However, there are many existing human uses that occur in the oceans, including commercial and recreational fishing, naval operations, maritime shipping, minerals extraction and general recreation. Marine spatial planning (MSP) tools can reveal tradeoffs between existing uses and the development of renewable energy sectors in the ocean, such as the potential effects of offshore wind energy (OWE) on existing human users and site activities.

Given the pressure to convert to renewable energy sources, demand for OWE development is strong on the U.S. East and West Coasts and globally [15]. The total global footprint of OWE farms is expected to increase 30% by the year 2028, compared to 2018 [16]. In the U.S., the Biden Administration has set a production goal of 30 GW of fixed-bottom OWE by 2030 [17] with an additional 15 GW of floating OWE by 2035 [18]. On the U.S. West Coast, Washington, Oregon and California have passed laws requiring utilities to transition to 100% renewable electricity sources by 2040 to 2045 [19]. Offshore wind energy will be a significant contributor in this transition: California has mandated 5 GW by 2030 and 25 GW by 2045 [20]; Oregon has a planning goal of 3 GW by 2030 [21]; and, while Washington does not have any OWE goals currently, the state government has shown strong support for the production and distribution of wind farm components [22]. Given this rapid expansion of OWE, MSP tools for investigating the tradeoffs between OWE siting and existing uses - which would also serve as a basis for impact mitigation - are in increasing demand.

Commercial and recreational fisheries are extraordinarily valuable resources with associated economies. In 2020, the United States commercial and recreational fisheries generated 1.7 M jobs, $253 B in sales and another $117 B in value-added impacts [23]. On the U.S. West Coast, gross revenue (ex-vessel landed) from commercial fishing averages (2011 - 2020) $739 million/year [24] with substantial linkages to local, regional, national and international economies, via tourism, the restaurant and retail groceries sectors and regional and international trade [23]. Commercial fishing is a major source of protein and micronutrients [25,26] in the U.S. and abroad, and demand is increasing with human population growth and depletion of other natural resources [27–29]; climate change exacerbates this natural resource depletion [30].

In the context of adding large-scale industrial development to an already crowded ocean, tradeoffs are inevitable; stewardship of public resources requires an understanding of these tradeoffs and how they are affected by management and policy [31]. There are very few studies that have investigated the tradeoffs between OWE development and commercial fishing. It is expected that development of OWE will cause potential loss of fishing opportunity and associated revenue due to exclusion from historical fishing grounds that are included within newly-developed OWE farms [32]. While there have been a handful of smaller scale studies that estimate fisheries value at a site or set of sites in areas subject to OWE development, these studies have not directly addressed potential tradeoffs between these two resource uses [33–35]. On the U.S. West Coast, previous comparisons of fisheries value and overlap with OWE development sites have been limited to Washington [36] and California [37,38] and have used relatively coarse-grained spatial data. In addition, while a case study tradeoff analysis has been done for an existing OWE lease area off the coast of California [39], such analyses between potential OWE and fisheries economics across the entire U.S. West Coast have not been done.

Existing assessments of potential OWE value on the U.S. West Coast have only evaluated a few specific sites and did not calculate potential economic implications of these activities [40]. Economic assessment in spatial planning has proven useful in Europe [41] because it identifies areas in the sea where conflict will likely be greatest based on financial motives in both industries, and facilitates socio-economic trade-off analyses during planning phases. While indicators of ecosystem condition and benefits [42] are often a key part of the multicriteria decision frameworks of ocean policy, they may also be challenging to combine into like terms and may not be representative of the tradeoffs affected industries would experience. For example, maps of fishing effort are often incomparable across fisheries due to incommensurate effort metrics (i.e., tow times for trawl gear vs soak times for fixed gear), and can misrepresent the importance of a fishing ground if a low value area has high effort. A recent literature review found that only 13% of ecosystem service studies actually quantified the impact of decisions on the environment, ecosystem services provision, and the values of those services to affected parties [43].

Here, we develop a coastwide geospatial analysis to assess potential overlap between OWE development and commercial fisheries and to examine economically relevant tradeoffs between these two ocean-use sectors. This type of analysis is useful for MSP related to OWE, or any other offshore development including aquaculture, in that it can investigate siting opportunities across the entire spatial domain, identify conflict between ocean uses before they occur, and be used to assess potential compensation should conflict arise [44,45]. This analysis advances prior MSP research that focuses on ecological or use patterns because it measures resource uses in units that are closer to the preferences and values of resource users and more broadly is at the frontiers of measuring the value of our ocean resources and cost-benefit analysis [46].

## Materials and methods

We generated spatially explicit models of wind energy potential and levelized cost of energy (LCOE) off the U.S. West Coast and overlaid them with maps of present value (PV) of economic activity from the commercial fishing sectors that account for the majority of fishing revenue. LCOE is an estimate of the average cost of producing a unit of energy over the lifetime of an energy project, given prevailing market conditions, technological capabilities, and wind resources. It was used in lieu of net present value as purchase agreements for wind energy on the West Coast have not been entered into yet. With these estimates in hand, we then spatially characterized patterns of overlap between OWE generation and fisheries. We examined overlap across the entire U.S. West Coast rather than at specific locations because spatial plans for OWE along the West Coast have been subject to frequent change, and that dynamism of potential site selection is likely to continue into the foreseeable future, particularly if demand for renewable energy accelerates. Finally, we look at tradeoffs between these uses using tradeoff frontiers for future wind energy development targets across the region. These estimates represent economic conditions prevalent currently and do not attempt to predict future changes in the economics or environmental conditions of either industry. All values are presented in 2020 U.S. dollars.

### Study area

We mapped fishing value throughout the full extent of the Exclusive Economic Zone (EEZ) of the West Coast of the United States, including within state waters (<5.5 km from shore). Offshore wind energy LCOE mapping was limited to water depths ranging from 60 - 1,300 m within the EEZ, per the Bureau of Ocean Energy Management’s (BOEM) current wind farm siting parameters and accepted norms for floating wind project siting [5,47]. While BOEM only has jurisdiction to do project siting in federal waters (>5.5 km from shore), we included state waters (<5.5 km from shore) in the wind modeling, subject to these other constraints. We delineated the boundaries of waters adjacent or associated with each state by extending the latitudinal state boundary lines from shore out to the extent of the EEZ, hereafter referred to as adjacent waters or water adjacent to “State Name”, regardless of whether or not any given location is within or beyond 5.5 km from shore.

### Data inputs

#### Potential OWE value.

We used the Integrated Valuation of Ecosystem Services and Trade-offs (InVEST^®^ https://naturalcapitalproject.stanford.edu/software/invest) OWE production (OWEP) model (v. 3.14.1 Workbench) [48,49] to create a map of LCOE for the entire U.S. West Coast. The model has been used in the U.S. and internationally for integrating OWE resources and values into ocean planning [50]. The model accounts for capital costs including a geospatially varying cost structure for transmission equipment, and other costs including maintenance, decommissioning, and more. Based on this and technical specifications of the turbine design and environmental context including long term wind speeds, it calculates the LCOE and an array of useful ancillary data. LCOE (in USD/MWh) is defined as “the unit price that would need to be received for energy that would set the present value of the project equal to zero” and is calculated using:


$$LCOE=\frac{\sum_{t=1}^{T}\frac{O\&M\cdot CAPEX}{{\left(1+i\right)}^{t}}+\frac{D\cdot CAPEX}{{\left(1+i\right)}^{T}}+CAPEX}{\sum_{t=1}^{T}\frac{E_{t}}{{\left(1+i\right)}^{t}}}$$

where *CAPEX* is the initial capital expenditures, *O&M* is the operations and management parameter, *D* is the decommissioning parameter, *E*_*t*_ is the annual energy produced in kWh,

*i* is the discount or Weighted Average Cost of Capital (WACC) rate (5.2%), and *t* is the annual time step, where *t* =  {1... *T*} [51]. Wind speed data for the region was sourced from the National Renewable Energy Laboratory (NREL) Wind Integration National Dataset toolkit [52]. The modeling assumed a farm design that includes sixty 15-megawatt (MW) wind turbines with a single transmission path to shore. Given the lack of existing large-scale floating OWE farms, we designed our model to emulate the likely infrastructure associated with planned projects [53]. Outputs are rasters where values for the given farm design are given on each grid point over the domain at a spatial resolution of 2.8 km. For calculations that depend on areal coverage, we transformed this by assuming a turbine spacing that corresponds to 3 MW of generation capacity per km^2^ [5,54] with a corresponding design wind farm size of 300 km^2^ and used weighted resampling of LCOE based on the InVEST estimate of energy generation per year per cell. We did not exclude areas in the OWEP that are currently considered off limits for OWE development, e.g., National Marine Sanctuaries or areas important for national defense. Our rationale was to provide as much information as possible to illuminate tradeoffs being made across all ocean areas including those currently considered off limits. Refer to S1-S3 Tables for model input and parameter details.

To contextualize planned future OWE we used development scenarios based on established and hypothetical projected OWE production goals for the corresponding state and the area that would be needed to meet those targets:

- California: 5 GW by 2030 and 25 GW by 2045 [20].
- Oregon: 3 GW by 2030 [21] and an assumed 15 GW by 2045 mirroring the targeted planned five times growth rate of California, in the absence of any explicitly defined existing targets.
- Washington: No goals as of May 2024, other than production of wind farm components [22], though interest has been expressed at the state and Tribal level. For demonstration of how this might interact with fisheries in waters adjacent to Washington, we used OR’s scenario of 3 GW by 2030 and 15 GW by 2045.
- Regional: A fourth scenario uses the aggregate targeted capacity across states for 2030 (11 GW) and for 2045 (55 GW) but relaxes the constraint that states’ targets need to be developed in state-adjacent waters and instead could be developed anywhere across the region.

#### Commercial Fishing Value.

To quantify the spatial distribution of commercial fishing revenue and better understand the potential exposure of fisheries to displacement by OWE, we generated 2.8 km resolution value maps for the species that account for the vast majority of total fisheries revenue landed off the U.S. West Coast. We used commercial fishing landing statistics for ports in Washington, Oregon and California [24] for all gear types from 2011 - 2020 to identify the top species, which account for ~ 84% of the total value landed. The top species, listed in order of gross revenue (percent of landings in parentheses), include:

- Dungeness crab (*Metacarcinus magister*, 30.9%)
- Pacific oyster (*Magallana gigas*, 8.2%)
- Pacific (whiting) hake (hereafter “hake”, *Merluccius productus*, 8.2%)
- California market squid (*Doryteuthis opalescens*, 7.9%)
- Geoduck (*Panopea generosa*, 6.0%)
- Ocean pink shrimp (*Pandalus jordani*, 5.9%)
- Pacific albacore tuna (*Thunnus alalunga*, 5.4%)
- Chinook salmon (*Oncorhynchus tshawytscha*, 5.1%)
- Sablefish (*Anoplopoma fimbria*, 4.2%)
- California spiny lobster (*Panulirus interruptus*, 2.2%)

We used the highest spatial and temporal resolution data available for each of the fishery sectors, which included state and federal logbooks, federal observer records, electronic monitoring, landings informed Vessel Monitoring System (VMS) data, and management statistics used by the Pacific Fishery Management Council (PFMC). All georeferenced data of individual towlines, pot strings and gear sets were filtered for activity erroneously occurring on land, traversing unreasonable distances or outside the typical depth ranges for the respective fisheries. See S4 Table for more details of data sources for each fishery species. We assigned a value to each individual fishing event (e.g., trawl towline, pot or trap string, etc.) by linking each activity to an associated port landed fish ticket provided by the Pacific Fisheries Information Network [55]. Fish tickets are individual receipts that fishers receive when they land a catch from a given fishing trip at a given port. Each fish ticket contains information on how much money the fisher sold their catch for (ex-vessel value), by species, weight, and price per kg. The unique fish ticket identification number is often reported in the fishing effort data sources, associated with each individual fishing activity record. This allows the fishing effort activity in the ocean to be linked to the value of the species landed shoreside. For those individual fishing events where a fish ticket was not listed, we calculated the fishing event value by multiplying the crew estimated weight of retained catch by the corresponding mean, weekly price per unit weight from fish tickets across all ports. All value calculations were done using a currency of U.S. dollars (USD), adjusted for inflation (AFI) relative to the year 2020 and were converted to PV of fisheries economic activity to better reflect the total economic contributions of these fisheries, not just the landed value (see Total Fisheries Economic Activity subsection below for more details). All maps displayed in this manuscript protect the confidential business information contained in raw fisheries monitoring data by excluding any grid cells containing data from fewer than three vessels. However, all analyses and summaries of those gridded data are based on the full dataset. We tracked the total number of unique vessels that landed catch for each fishery to provide a general sense of the fleet size associated with each fishery. Finally, we calculated the value of each fishery per km^2^ for each of the three states for generally comparing the value per unit area of these fisheries. See S4 Table for more details on each targeted species analyzed.

We used state provided commercial fishery logbook data for Dungeness crab that was landed at Washington and Oregon ports, and landings-informed VMS data for Dungeness crab landed at California ports. Logbook data are provided by individual fishers, which includes the geocoordinates of the start and end of each pot string they set and when the string was retrieved. California does not have a logbook program for their Dungeness crab fishery, so we linked fish ticket data to corresponding VMS-monitored fishing trips following the approach of [56,57].

Since all Pacific oyster culture occurs within bays and estuaries, and at depths shallower than 5 m [58,59], overlap with potential OWE was presumed to be zero.

We used data from the West Coast Fisheries Observation Science Program (WCFOS, [60,61]), which consists of the At-Sea Hake Observer Program (A-SHOP) and the West Coast Groundfish Observer Program (WCGOP), as well as electronic monitoring (EM) data for both the shoreside and at-sea sectors [62]. Observers in the WCFOS Program record the set and retrieval latitude and longitude for each haul based on the captain’s logbook. EM data includes the same data recorded by both the EM system itself and by the captain in their logbook; we rely on the EM system’s records whenever possible. We analyzed the shoreside processing and at-sea processing sectors separately to reflect differing industry interests across the fleets.

We used California Department of Fish & Wildlife (CDFW) state logbook data that reports the geocoordinates of each California market squid fishing event. Each logbook entry lists the associated PacFIN fish ticket ID, which we used to assign a value to each entry. We then overlaid all of the point geocoordinates on the 2.8 km OWEP grid and calculated a value for each OWEP grid cell.

Since all geoduck fishing on the U.S. West Coast occurs within Puget Sound, Washington, overlap with potential OWE was presumed to be zero.

We used state-provided logbook data for pink shrimp that was landed in Washington and Oregon ports. A small proportion of coastwide pink shrimp landings occur in California, but the associated logbook data were not available for this analysis.

Commercial fishing vessels and recreational charter fishing vessels fishing for albacore tuna are required to record catches of highly migratory species, effort, and other data in logbooks and report this information to the National Marine Fisheries Service’s Southwest Fisheries Science Center [63]. We used the recorded geocoordinate information for each fishing event (i.e., troll or hook-and-line set) for the commercial portion of the albacore tuna fishery, which represents all commercial albacore tuna fishing that occurs within the EEZ of the U.S. West Coast. The geocoordinates recorded in these logbooks had unusually regular spatial patterns (e.g., fishing locations show up in a gridded manner versus a relatively random manner that would be expected); we used statistical resampling tools to remove these biases in location reporting. Final geocoordinates were overlaid on the 2.8 km OWEP grid and we calculated a value for each OWEP grid cell.

We used data for the commercial Chinook salmon fishery provided by the PFMC [64]. The PFMC produces statistics each year based on fish ticket and fishing effort data held and managed by Pacific States Marine Fisheries Commission. These statistics include the number of ocean caught Chinook salmon, by month and fishery region. We linked data reported by the management region to the corresponding region in the geospatial data based on the PFMC regions [65,66]. We then multiplied the number of fish landed in each region by the corresponding average dressed weight data and multiplied that by the corresponding mean weekly landed value per kg to assign value. Of all the fishing activity data sources that we used, these data had the coarsest spatial resolution. We refined the PFMC fishing regions further by subdividing into shallow (0 -100m) and deep (100-200m) areas, based on analyses of spatially explicit fishing location data found in two previous analyses [67,68].

Similar to the hake data, we used federal observer program [60,69] and EM data for the sablefish fishery, which reports the start and end geocoordinates of each trawl towline or fixed gear set [62]. Sablefish are caught in both bottom trawl and fixed gear (pot and longline) fisheries across the U.S. West Coast. Sablefish are not targeted by the at-sea processing fleet, so only WCGOP and EM data were used for this fishery. Observers record the set and up latitude and longitude for each haul (trawl towlines and pot and longline sets) based on the captain’s logbook. EM data includes the same data recorded by both the GPS of the EM system itself and by the captain in their logbook; we rely on the EM system’s records whenever possible.

We linked the landed value of California spiny lobster reported on each PacFIN fish ticket with the unique CDFW fishing block reported on the fish ticket. CDFW fishing block polygons [70] are 10-arcminute resolution (~18.5 km at the equator). We then overlaid the 10-arcminute fishing blocks with the 2.8 km OWEP grid and calculated a value for each OWEP grid cell.

##### Expansion of grid cell values

Most of the fisheries we examined do not have logbook or geocoordinates for 100% of fishing events, so we applied expansion coefficients on a grid-cell-by-grid-cell basis to approximate the spatial distribution of all fishing value represented in the comprehensive PacFIN commercial fishing port level landings database. This type of expansion or correction of fisheries activity data is commonly used by agencies who collect these data [71] and by other researchers who have used these data [72,73]. After we calculated the value of each grid cell for each fishery species in each year using fishing event geocoordinates, we summed the value of all grid cells for that fishery for each year and compared with the total landed value of that fishery for the corresponding year present in the PacFIN database. We then applied a correction factor to each grid cell for each year so that across grid cells in any given year, the total was equal to the landed value for the species in that year according to the PacFIN database. For example, if a given fishery species in a given year had 50% of their landed value represented across recorded fishing events, the value in each of the grid cells for that year was divided by 0.5. See S5 Table for the expansion coefficients we used for each species.

##### Total fisheries economic activity

Since impacts of fishing on community-wide economic activity may also be an important policy consideration, we calculated the economic activity for each targeted species using multipliers derived from the input-output model for Pacific Coast Fisheries (IO-PAC; [74]). Within a given port group, IO-PAC multipliers account for upstream and downstream economic activity resulting from fishing activities. Upstream activity includes purchases of fuel, ice, and supplies that serve as inputs into fishing effort, and downstream activity includes seafood processing, transportation, and storage. Induced expenditures as a result of crew and captain wages are also accounted for in these multipliers. They are calibrated using a variety of data, such as cost-earnings surveys [75–77]. These data also include patterns of consumption for specific geographic regions from Impact Analysis for Planning software [78] and fish ticket data from PacFIN describe historical catch patterns [55]. Notably, IO-PAC does not estimate economic welfare, does not verify whether benefits exceed their costs, and assumes individuals do not substitute or adjust to changes.

IO-PAC data inputs range from 2014-2020, depending on availability; refer to S6 Table for additional details and the specific multipliers that we used for calculating total economic activity associated with each targeted species. Multipliers were applied to each mapped grid cell based on species and the corresponding state where the fishing occurred.

We calculated the PV of fisheries economic activity for each species by first dividing inflation-adjusted total economic activity from 2011–2020 by 10 (mean annual economic activity) and then applying a 5.2% discount rate [79] to this mean annual economic activity over a 33-year period (most common length operating period quoted on recent BOEM lease sales). We do not combine these monetary estimates of OWE and fisheries as they are not in common terms. Power purchase prices and reliable multipliers for floating OWE are not available, given the nascent state of this industry in the U.S. As wind farms begin operating in the near future, representing these industries in like terms will be an informative next step.

### Comparing OWE and commercial fishing value overlap and tradeoffs

We characterized the degree of potential overlap between OWE farms and fishing activities on a grid-cell by grid-cell (2.8 km) basis using two measures. First, we generated overlap index maps by dividing PV of fisheries economic activity by the corresponding LCOE for each grid-cell. Second, we calculated the Spearman’s rank correlation (\rho) between each fishery and LCOE across the study area.

In the context of identifying optimal locations for OWE development, a planner may want to minimize both the LCOE and the exposure of fishing revenue. Doing so efficiently uses limited offshore resources and enables planning in accordance with the private incentives of the stakeholders involved. These values are not additive, so exploration of siting strategies relies on suitability across two dimensions. Multiobjective optimization is a useful tool for sorting out which sites meet both goals and may be prioritized for wind energy development. The optimization is done under a constraint that total OWE development must be sufficient to meet an energy production target. Optimizing yields sets of sites that are Pareto optimal - meaning that in our case, LCOE could not be decreased without increasing the exposure of fishery value, or vice versa. This optimization yields many optimal sets of locations for a given development target and all of these sets form a tradeoff frontier. Plotting this frontier shows tradeoffs between objectives for optimal sets and can be helpful in choosing among them [80], though the actual final choice between these optimal sets is not a direct output of these methods.

For tractability and realism in the optimization problem, the grid was resampled (upscaled) to ~ 300 km^2^ for both LCOE and fisheries PV by aggregating the 2.8 km modeling grid to 6 by 6 (17.1 x 17.1 km) groups of grid cells. This reduced the search space of the optimization algorithm while also constraining the model to searching over wind farm-sized squares. The NSGA-2 multi-objective evolutionary algorithm was used from the R package “mco” (version 1.16) for optimization [81]. The output of this algorithm is a tradeoff frontier of Pareto optimal sets of wind energy sites that meet a specified development target that tracks which wind farm grid cells were chosen in each optimal set. We generated maps illustrating which grid cells were selected for a given point along the various optimization frontiers, in order to demonstrate spatial patterns in wind farm siting across three optimal choices across the frontiers: fisheries centric, balanced tradeoff between fisheries and wind, and offshore wind centric, corresponding to the 10^th^, 50^th^ and 90^th^ percentiles along each Pareto frontier. We generated the maps for optimizations run at both the region-wide and state-wide (constrained to the waters adjacent to the corresponding state/target capacity) scales, and for 2030 and 2045 OWE capacity targets. We also present an analysis that averages over the Pareto sets along the frontier for each development target, to help demonstrate the unconditional expectation of impacts on different fishery species across all optimal site sets [82,83].

## Results

### Offshore wind energy

**Fig 1:**
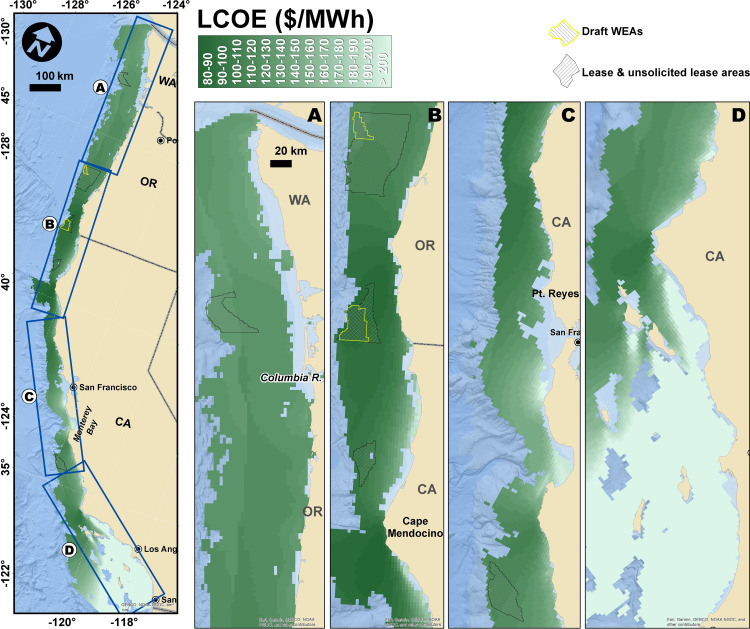
Map series of levelized cost of energy (LCOE, 2020 USD per megawatt hour) of OWE off the U.S. **West Coast predicted by the InVEST OWEP model.** Darker green grid cells have lower cost energy production, which would be more profitable for development, all else equal. Cross-hatched regions are prospective or currently leased OWE call areas. Basemap reprinted from World Ocean base under a CC BY license, with permission from Esri, original copyright © 2024. Basemap content is the intellectual property of Esri and is used herein with permission. Copyright © 2024 Esri and its licensors. All rights reserved.

The modeled LCOE varied considerably across the region, with the lowest cost grid cell being $81/MWh and the highest being over $800/MWh (Fig 1). By state, California had the highest mean LCOE at $186/MWh (±$116 sd), but had higher variance over a larger area compared with the other two states. Oregon had the lowest mean LCOE at $114/MWh (±$11 sd), and Washington was slightly higher at $127/MWh (±$4 sd) with very little variation across all potential sites.

### Commercial fishing

**Fig 2:**
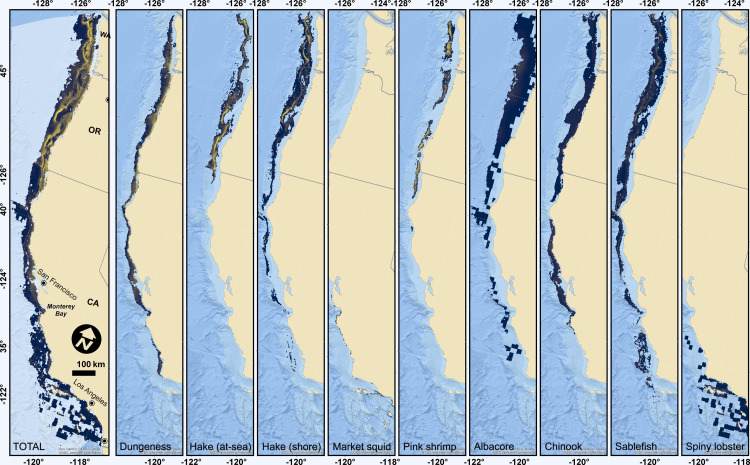
Maps of the overlap index for all fisheries species analyzed (far left map) and for each of the individual species. Light blue region on the far left map indicates the exclusive economic zone (EEZ). Mapped overlap indices share the same scale across each species and for the combined species, making maps directly comparable. Overlap index is the grid cell by grid cell division of fisheries present value with economic activity (AFI **M** USD re. 2020) and LCOE of OWE. Grid cells with high overlap indices have high fishing value and low OWE production costs, whereas grid cells with low indices have either low fishing value or high OWE production costs, or both. Basemap reprinted from World Ocean base under a CC BY license, with permission from Esri, original copyright © 2024. Basemap content is the intellectual property of Esri and is used herein with permission. Copyright © 2024 Esri and its licensors. All rights reserved.

Commercial fishing operations for the eight target species we studied occupied a vast area off the West Coast, 572,596 km^2^ (S1-S10 Figs), which covers about 70% of the entire West Coast EEZ. The vast majority of the EEZ off Oregon and Washington was fished in the last 10 years (99.1% and 87.7%, respectively), while more than half (56.9%) of California’s EEZ waters were fished (S1 Fig). The actual, realized footprint of fishing operations is most certainly larger than that shown on the map, as we did not include areas used for transit by fishing vessels and we did not include fishing events that did not catch any fish. Bottom trawl fishing was restricted to depths less than 1,300 m coastwide, with additional state and federal spatial closures off of California (S1 Fig; see also [84,85]). Therefore, areas where bottom trawl fishing is allowed overlapped directly with areas of high wind energy potential, coastwide and especially off of Washington and Oregon (Fig 2 “sablefish”, and S9 Fig). The total present value (PV) of fisheries economic activity for the eight species that we analyzed was $31.096 B over the next 33 years, and 3,778 different vessels fished for the eight species analyzed. See the Supplementary Results section in the supporting information document for additional details for each of the individual species examined and refer to Table 1 for summary information on the total value, value per km^2^, number of associated vessels and depth ranges over which each species is fished.

**Table 1:** Present value (PV) with economic activity (2020 USD) for entire fishery and per unit area for each fishery, number of associated vessels and fishing depth statistics associated with each of the fisheries analyzed over the 10 years spanning 2011–2020.

Fishery	Total Value (B USD)	Value (K USD/km^2^)	Vessels (n)	DEPTH (m)		
				Mean	SD	Range (1st - 3rd quartile)
**Dungeness crab**	10.792	230.6	1,620	46.2	41.41	19 to 63
**Hake (at-sea)**	5.157	105.3	36	718.8	670.80	378 to 687
**Hake (shoreside)**	3.137	60.6	120	379.9	209.17	203 to 501
**Market squid**	2.738	268.0	242	62.8	223.04	26 to 43
**Pink shrimp**	3.203	168.2	166	145.4	44.37	124 to 158
**Albacore**	2.132	4.2	1,108	2,134.8	836.47	1,512 to 2,839
**Chinook salmon** ^*^	1.126	22.2	1,875	87.0	43.18	59 to 109
**Sablefish**	1.852	20.6	989	478.2	239.96	340 to 594
**Spiny lobster**	0.960	13.1	368	64.5	202.29	12 to 33

*Based on geolocations reported in published Chinook fishery papers [67,68], overlaid on high resolution bathymetry grids [57].

#### Overlap between OWE and commercial fishing.

##### General patterns of fisheries overlap with wind energy.

The overlap index (fishery PV divided by LCOE within the footprints of commercial fisheries) varied by species, gear type, and state (Fig 2). Looking across all target species in total, there was generally some fishing activity across the entire potential OWE domain north of Monterey Bay, California (Fig 2 “TOTAL”). As such, most fisheries, other than spatially expansive albacore, and spiny lobster and market squid (which generally operated at depths < 100m, Table 1), had their entire footprint within the region where OWE might be developed (Fig 2). The intensity of potential overlap indicated by the index also varied by species and geographic location. Across all species, the highest overlap indices (yellow regions) were concentrated off the coasts of Oregon and Washington, and northern California (Fig 2 “TOTAL”). Pink shrimp and both at-sea and shoreside hake had distinct bands of high overlap indices, and sablefish had similar distinct bands off Oregon and Washington, though not as intense (Fig 2).

Based on the Spearman’s rank correlation, there was a significant negative correlation between the PV of all species combined and LCOE, and with hake (at-sea), pink shrimp, albacore and sablefish (S7 Table). This negative correlation indicated significant overlap in the highest value fishing grounds with areas expected to have lower LCOE, and presumably higher OWE profit. Conversely, there were significantly positive correlations between LCOE and the PV of Dungeness crab, shoreside hake, market squid, Chinook salmon, and spiny lobster (S7 Table), which suggested lower potential conflict between fisheries for these species and OWE development.

#### Levelized cost of energy in areas where fish are caught.

**Fig 3:**
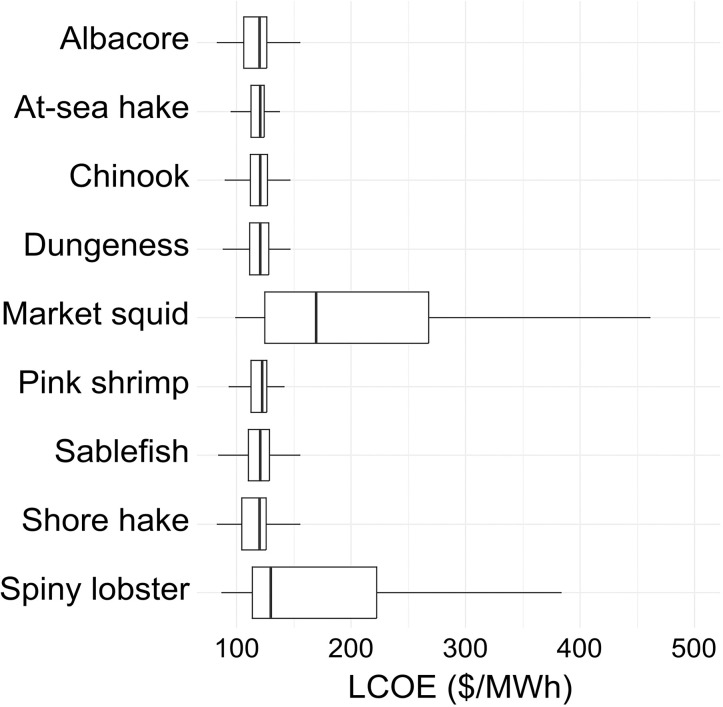
Boxplots of the levelized cost of energy (2020 USD per megawatt hour) in the areas where different fishery species are caught across the U.S. West Coast.

Understanding where OWE development will take place across the region relies in significant part on LCOE since lower cost energy production is more profitable, all else equal. The distribution of LCOE varied significantly across space (Fig 1) and within the spatial footprints where different fish are caught (Fig 3). The median LCOE values were similar across locations where most species were caught with the exceptions of market squid and spiny lobster, which were caught in areas with higher LCOE and presumably lower incentives for OWE development (Fig 3).

#### Tradeoffs between LCOE and fishery value.

**Fig 4:**
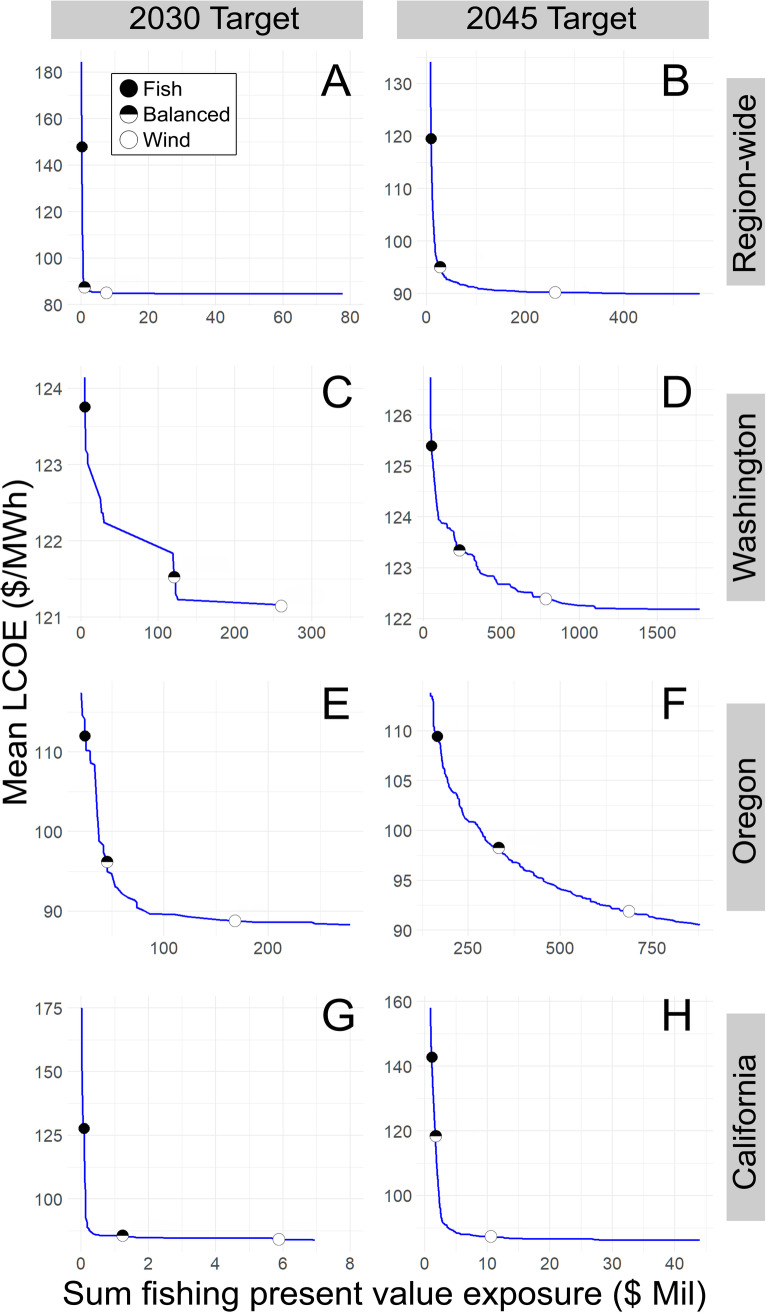
Tradeoff frontiers between levelized cost of energy (LCOE, 2020 USD per megawatt hour) and fishing present value (present value, 2020 millions USD) for different OWE development scenarios. The frontiers represent Pareto optimal siting choices to achieve the target level of development. Each of the three labeled points along each frontier represent the 10^th^, 50^th^, and 90^th^ percentiles for each Pareto frontier, corresponding to fisheries centric (fish), balanced and offshore wind energy (wind) centric optimal choices, respectively. Panels (A) and (B) represent a regional approach towards meeting the combined regional target across states for 2030 (11 GW capacity) and 2045 (55 GW capacity), respectively. Panels (C), (E), and (G) represent state-based approaches for Washington, Oregon, and California respectively where they are constrained to meeting their OWE development targets for 2030 in state-adjacent waters. Panels (D), (F), and (H) represent state-based approaches for 2045 capacity targets.

Fig 4 presents tradeoff frontiers for the cost of wind energy generation and the corresponding exposure of fishery value that would be displaced. Frontiers correspond to OWE development targets in 2030 (panels A, C, E, G) and 2045 (panels B, D, F, H). The state-based tradeoff frontiers for 2045 (Fig 4D, F, and H) were qualitatively similar to their 2030 results. Points along the frontier represent feasible and optimal development options to meet the development targets, and selection among points on the frontier requires either increasing fishing PV exposure or wind energy costs. Very steep or flat sections of the frontier exhibit site choice tradeoffs that may be easy to make. For example, in Fig 4G a very small increase in the exposure of fishing revenue yielded a dramatically lower cost of energy production, while in Fig 4C a small increase in energy production costs reduced the exposure of fishing values to almost zero. Making decisions in these cases may be easier than when movement along the frontier results in significant tradeoffs in both sectors, as exemplified in Fig 4B where reducing the cost of energy production below $100/MWh came with a significant loss of fishing value.

For 2030, California had several sets of optimal choices in adjacent waters that minimize LCOE with very little exposure to fishing PV (Fig 4G), partially reflecting lower fishing effort in waters off of California. The existence of more sites of this type in California-adjacent waters than are needed to meet its target means that optimal regional planning for 2030 would generally site most development in waters adjacent to California - hence the similarity between panels A and G. Fishery PVs are far more exposed in Oregon and Washington than in California, and Oregon tended to have lower LCOE than Washington. If Oregon’s target was met exclusively by developing in state-adjacent waters, there would be significant tradeoffs between LCOE and exposed PV (Fig 4E). In Washington, the range of LCOE was limited across optimal sites, so the tradeoff choices would be easier to make given that it is only a difference of ~ 3% of LCOE to reduce fisheries PV exposure from over $100 million to close to zero (Fig 4C).

For 2045, the greater scale of development meant tradeoffs were inevitable even at the regional scale (Fig 4B), as reflected by the less convex tradeoff frontier versus 2030. California waters up to 25 GW of development still had generally low LCOE sites with low fishing PV exposure (Fig 4H), but adding another 30 GW on top of that at the regional scale resulted in having to add areas with higher fishing PV exposure across the region to keep LCOE low. More sites were chosen from Washington and Oregon among the optimal sets than in 2030 (Fig 4D and F).

**Fig 5:**
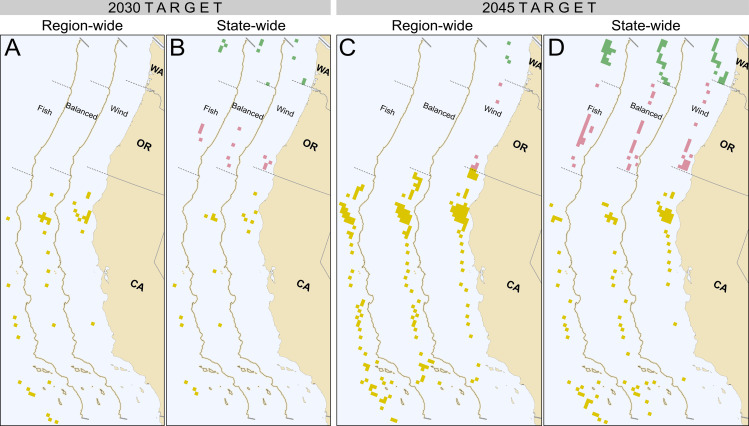
Maps of wind area grid cells that were selected at three example points along tradeoff frontiers. Each of the three sub-maps within the four map panels A, B, C and D show wind farm grid cells that were selected at three example points along each frontier in Fig 4 representing the 10^th^ (fisheries centric, “Fish”), 50^th^ (“Balanced” between fisheries and wind), and 90^th^ (offshore wind centric, “Wind”) percentiles for each Pareto frontier. Green, rose and gold grid cells correspond to the waters adjacent to the states of Washington, Oregon and California, respectively. Map series in panel (A) indicate wind farm grid cells that were chosen based on a regional (coastwide) approach towards meeting the combined targets across states for 2030, and panel (B) is from a state-based approach for 2030. Map series in panel (C) show wind farm grid cells that were chosen when state targets were developed based on a regional approach towards meeting the combined targets across states for 2045, and panel (D) is from a state-based approach entirely in waters adjacent to each state for 2045.

Delving further into the aggregate spatial results just discussed, Fig 5 presents maps of the grid cells that were selected at three points across each frontier that show the variation in siting for optimal choices with different priorities: fisheries centric (“Fish”), balanced tradeoff between fisheries and wind (“Balanced”), and offshore wind centric (“Wind”), which are the 10^th^, 50^th^ and 90^th^ percentiles across the frontier, respectively. Regional optimization avoids siting wind in 2030 (Fig 5A) and 2045 (Fig 5C) in Oregon and Washington except when selecting to prioritize lower wind costs in the 2045 scenario (Fig 5C “Wind”). Prioritizing wind at the regional level emphasizes development off of Northern California in 2030 (Fig 5A “Wind”) and 2045 (Fig 5C “Wind”), and moving towards a balanced or more fishing-oriented prioritization spreads development out along the California coast (Fig 5A “Fish” and “Balanced”; Fig 5C “Fish” and “Balanced”). Optimization of the various state targets when limited to the state-wide scale (waters adjacent to each state) for Oregon and Washington tends to select more southerly areas when prioritizing for wind (Fig 5B and 5D “Wind”), compared with fishing prioritization (Fig 5B and 5D “Fish”).

**Fig 6:**
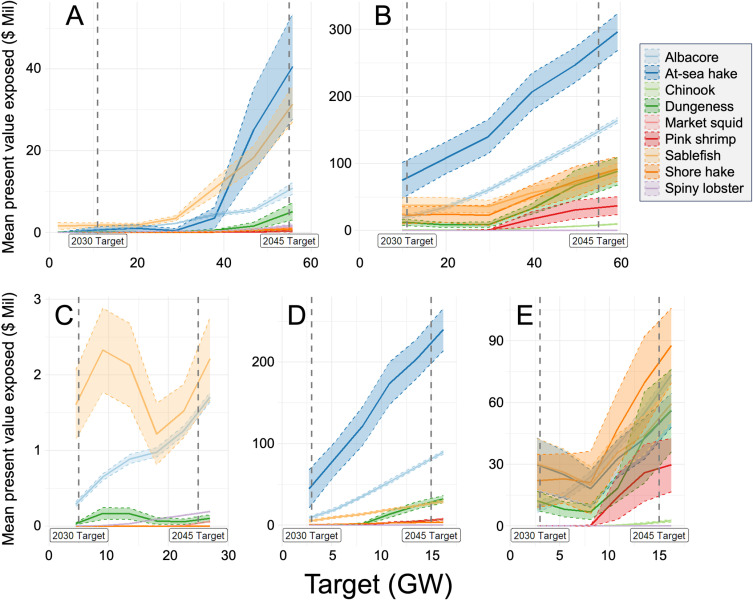
Fishery species’ exposure (mean present value exposed by fishery, 2020 millions USD) across Pareto optimal sites for different levels of OWE development (Target, gigawatts). 95% confidence intervals are given in corresponding shaded color to species’ trendlines. Targets labeled 2030 and 2045 indicate goals or mandates by individual states (panels (C), (D), and (E)) or regional aggregate goals (panels (A) and (B)). Panel (A) represents an unconstrained optimization, where aggregate development targets across all three states are met by siting OWE in waters anywhere within our modeling domain. Panels (C) - (E) represent constrained optimization at the state level for California, Oregon, and Washington, respectively, where their individual targets are met only in state-adjacent waters. Panel (B) represents the sum of panels (C) - (E), where development is summed proportionately based on the relative targets - i.e., 11 GW represents 3 GW each from Washington and Oregon and 5 GW from California.

While Fig 3 presents LCOE by fishery to help understand which fisheries overlap with areas most suitable for OWE, it does not provide a sense of the scale of their potential exposure or how that may change as development expands through time. Fig 6 presents fishing PV exposure by averaging over Pareto optimal sets of sites when planning regionally (panel A) or when development targets are constrained to the waters adjacent to each state (panels B - E), for development targets up to 2045. This provides an unconditional expectation of potential impact by fishery species, as if the actual siting choice is done randomly from the optimal sets of sites. Importantly, this calculation weights tradeoffs equally between LCOE and fisheries PV and actual exposure to fisheries would be significantly higher if siting choices were not optimized or if LCOE were prioritized more than fisheries PV in actual decisions, or vice versa.

For 2030, albacore, sablefish, and at-sea hake were the most exposed by development across optimal sites (Fig 6A - 6E). Scaling up development to 2045 targets featured all of the same species as most exposed with the notable addition of Dungeness crab to the group. Other target species had lower exposure, with the lowest being market squid. Variation in the ranking of species and the nominal value of exposure is considerable when planning development as constrained to the state level, which is reflective of current management of OWE development on the West Coast. Oregon clearly had the most fishery species’ exposure as OWE development advances (Fig 6D) compared with California (Fig 6C) and Washington (Fig 6E). Note the range of the y-axis for Oregon is nearly 100 times that of California and over twice that of Washington. Exposure to at-sea hake was notable in Oregon and drives aggregate regional exposure of this species (Fig 6A and 6B). Figure 6B, representing the summed regional exposure if siting was optimized at the state level, is mostly driven by exposure originating in Oregon and to a lesser extent Washington, with almost no exposure coming from California. Comparing Fig 6B and 6A, which differ only in the imposed constraint of state-level optimization, shows the benefit of planning regionally with aggregate regional fisheries exposure decreasing roughly eightfold versus planning at the state level for 2045 targets. S11 Fig presents these results as a percentage of total per-species fishing revenue, with similar qualitative interpretation.

## Discussion

Development of OWE is expanding at a rapid pace globally, and here we present an application of marine spatial planning tools that can be helpful for proactive planning for siting purposes at the regional scale in terms that are economically meaningful to the involved sectors. The spatial overlap analysis highlights which regions and fisheries are likely to have the most conflict across the region. The tradeoff analysis shows how to optimally choose where to site for different OWE development scenarios and the extent of tradeoffs between wind energy and fisheries, and facilitates planning to meet either region-wide or state-specific OWE development targets. The analysis also identifies which fishery sectors are likely to be the most exposed as OWE development expands. Based on the coastwide 2030 buildout goals or mandates, most target species have relatively low exposure, with albacore, at-sea hake and sablefish having the most exposure. Looking at the most ambitious goals or mandates by 2045, we found that fisheries targeting those same species and the lucrative Dungeness crab had elevated exposure, with the least exposure for the market squid fishery. We also show that planning regionally for OWE, at least in terms of fisheries exposure, is preferable to individual states attempting to meet their development targets in their adjacent waters. In this discussion we touch on three key points related to this research: first we describe how our analytical products could be applied to planning, mitigating and compensating for OWE development; second, we discuss the importance of indirect effects from OWE development; and, third, we describe future directions for research to better account for continuing impacts of climate change.

### Application to planning, mitigating and compensating

Our analysis provides a broad-scale, fishery-wide perspective to the overall marine spatial planning process for OWE development. It also underscores the potential pitfalls of managing OWE development at the state level based on corresponding state goals or mandates. For example, based on 2030 planning goals, if Oregon’s capacity goal is met only from within its adjacent waters, there will be significant tradeoffs between LCOE and exposed fishery PV. However, these tradeoffs could be reduced if Oregon were allowed to access portions of California or Washington adjacent waters to meet its state planning goals. A regional approach towards developing wind where states coordinate to meet their development targets could reduce fishing exposure. States on the east coast are coordinating on power purchasing already, and coordinating on siting could be a way to meet targets while controlling externalities [86]. In-depth investigation of OWE transmission infrastructure and grid scenarios are underway for the Pacific coast and have already been completed for the Atlantic coast [87] that extend beyond the direct-to-shore shortest distance cable routing assumed in this analysis. Integrating the lessons between these projects would be a logical next step for improving regional planning.

One of the goals in the development of this new ocean-use sector is to avoid, minimize, and mitigate potential impacts to environmental resources and existing ocean users [88]. The data analyzed here have already contributed to the identification of ‘suitable’ areas for OWE development in the Coos Bay and Brookings Call Areas off of Oregon by identifying the relative importance of subcomponents of these areas to West Coast fisheries. The resulting draft Wind Energy Areas (WEAs) largely avoided and minimized areas with the highest levels of importance to fisheries, while maintaining areas of necessary size to meet the stated ocean energy goals for the state of Oregon [89]. The results of this analysis expand our understanding of the extent to which avoidance strategies are possible across the entire West Coast given the distribution of resource values for both sectors.

Avoiding impacts on existing marine uses is the ideal first step in MSP related to OWE development. However, when impacts from OWE development cannot be avoided, the general strategy is mitigation. Mitigation can include modification to OWE construction and operations and/or to fishing technologies that reduce impacts from wind farms. In situations where mitigation proves challenging, compensation to existing ocean users may be required for impacts related to OWE construction and operations [45]. To this end, this analysis provides methods and data for assessing fishing value exposure by area and by fishery. Approaches for determining compensation in the context of OWE development in the U.S. are not yet fully developed [90]. Initial compensation plans have involved ad hoc processes for individual wind farms with varying approaches. A coalition of states [91,92] on the Atlantic coast is attempting to create a regional framework for standardizing the process of estimating impacts as well as collecting and distributing compensation funds from developers. Federal policy has been proposed as well [93], but requires congressional action. These efforts are challenged by the conventional issues associated with establishing efficient and agreeable contracting mechanisms for externalities, as well as scientific gaps in understanding ecological and economic impacts. Currently established compensation strategies in Europe reflect this reality as they vary widely across countries and include factors such as who negotiates, whether compensation is based on net or gross fishing revenue impacts, the duration of responsibility, when payments are made, whether navigational costs are included, and more [94]. Establishing a perimeter around impacts and affected parties to identify standing is also part of the process. Fisheries are embedded in coastal economies and their economic impact and cultural importance extend well beyond affected fishers to other businesses and into the broader community.

### Fishing impacts

This analysis has focused on assessing the value of fisheries and the cost of OWE production, and then using that information to investigate the direct tradeoffs in terms of the potential exposure of commercial fisheries to OWE development. However, the ecosystem impacts of hydroelectric development in the U.S. and associated loss of ecosystem services [95] provides a cautionary tale of the pitfalls associated with overlooking indirect effects of infrastructure projects with long design lifespans. The nascency of large-scale OWE development necessitates adaptive and responsive monitoring of the indirect impacts of decreasing fishing activity [44,96]. Three such potential indirect impacts of OWE development warrant discussion here, given their significance: seafood trade and transfer effects; reserve effect; and marine circulation.

If the displacement of fisheries due to OWE development in the U.S. reduces domestically landed and consumed catch, reliance on foreign seafood imports may increase and raise megafauna bycatch rates outside the U.S., i.e., transfer effects [97]. However, the ecological effects of fisheries displacement from OWE areas on target species abundance and availability to fisheries is not yet clear. The consensus amongst many fishers is that OWE facilities will completely exclude all fishery sectors, essentially reducing the total footprint over which fisheries operate with commensurate potential negative impacts from OWE infrastructure (e.g., EMF, noise, etc.) on target species [98]. However, spatial fishery closures do not always result in decreased revenue for or exclusion of fishers [94,99]. OWE facilities may even create a reserve effect that would enhance or redistribute target species that could actually benefit commercial fisheries [100–102]. However, the potential for this reserve effect will depend on many factors. For example, fishing behavioral responses can be complex and nuanced, and in some cases counterproductive from a stock status perspective [103]. In addition, wind farms may affect coupled biophysical dynamics in the ecosystem, including wind patterns leeward of wind farms [101], upwelling [104] and associated ecosystem processes that drive many target species’ population dynamics and spatio-temporal distributions. Our analyses do not capture these additional complexities, and future research that evaluates them will be fruitful [105,106].

### Future directions

An important limitation of this study is that we did not link fishing value in each grid cell with the associated port where the catch was landed. Even in fisheries where there was little overlap with potential OWE development, e.g., market squid, overlaps that occur will be port- and fishery-specific and will affect someone or some port. There are about 70 ports that actively land commercial fisheries on the U.S. West Coast, which would be the finest level of detail that could be discerned in terms of OWE impacts on recipient fishery communities. Future efforts to do this on the West Coast could link impacts of OWE back to macroeconomic impacts across communities that are directly and indirectly involved in fishing to understand the reach and economic activity exposed to OWE development at any location within the EEZ [33]. This work also could incorporate spatially explicit fishery operating cost estimates, to better understand how costs affect the spatial distribution of net economic value. Ideally, future analyses will compare wind energy and fishery value estimates in common terms, as power purchase agreements are signed and pricing information allows for the calculation of net present value of wind energy.

Our analyses are an historical snapshot in time, representing the present value of the ocean for the most lucrative fisheries operating off the U.S. West Coast between 2011 and 2020. However, climate change is predicted to alter the spatio-temporal distributions of marine resources, including many of the fisheries’ targets described in our results [107,108]. Changes in these species’ distributions will likely alter the concentrations of fishing effort in the future and the accessibility of these species to fishers [109,110]. For example, California market squid have been one of the most conspicuous species observed off the coast of Oregon in recent years owing to ocean warming [111,112], and their apparent poleward redistribution was qualitatively evident in our landings data. They were virtually absent in Oregon landings for the first five years of the dataset, but increased substantially for the last three years. In addition, one of the most valuable groundfish species, sablefish, is predicted to shift further offshore into deeper waters as bottom temperature and dissolved oxygen concentrations change, although this shift is not consistent and varies by latitude across the West Coast [108]. A species not included in these analyses, Pacific sardines (*Sardinops sagax caerulea*) had historically been in the top 10 fisheries in terms of revenue, but this fishery was closed in 2015 when the populations collapsed [113]. Sardine populations can be highly variable owing to oceanographic conditions and food availability, and they are projected to return to historic abundance and harvest levels by mid-century [114]. Incorporating the projected impacts of climate change (e.g., stock fluctuations and shifts in species distributions over the next century) may help identify and mitigate changes in risk and vulnerability of individual fishing sectors, ports-of-delivery and coastal communities via interactions between OWE development, climate change and lost future fishing opportunities [115]. Finally, wind resources themselves may be affected by future climate change through spatial shifts in the intensity and persistence of wind. Analyses specific to the U.S. are lacking, but modeling efforts in Europe [116,117] and China [118] suggest that some areas will experience decreased wind velocity under various climate change scenarios whereas others will be unaffected or have increased wind velocities. OWE farms are durable assets with lifetimes that extend over 30 years, so these dynamics should inform projected future revenues and siting choice implications for both the current day and the near future as we strive to meet development goals or mandates.

## Conclusions

The development of OWE in the U.S. and elsewhere faces significant headwinds, given conflicts with existing resource extraction activities and other economic activity in shoreside communities, gaps in research at relevant scales in decision-relevant metrics to understand environmental and social effects of OWE implementation [119], and volatile supply chain and fiscal solvency problems [120]. The analysis here attempts to address these gaps by providing decision-relevant information at scale to help assess tradeoffs between users of public resources. The results suggest there is significant spatial heterogeneity in wind and fisheries value and that thoughtful consideration of tradeoffs in stakeholder relevant metrics can provide critical decision support information. Looking at the historical activity of existing resource users is an important step to enable thoughtful advance planning of economic tradeoffs between OWE implementation and fishing communities.

## Supporting information

S1 File S1 Fig. Map series of present value with economic activity (2020 millions USD) of the combined fisheries studied off the U.S. West Coast. Yellow outlined cross hatched regions are Draft Wind Energy Areas (WEAs) and black outlined cross hatched regions are prospective or currently leased OWE call areas, or unsolicited lease areas. Basemap reprinted from World Ocean base under a CC BY license, with permission from Esri, original copyright © 2024. Basemap content is the intellectual property of Esri and is used herein with permission. Copyright © 2024 Esri and its licensors. All rights reserved. S2 Fig. Map series of present value with economic activity (2020 millions USD) of the Dungeness crab fishery off the U.S. West Coast. Yellow outlined cross hatched regions are Draft Wind Energy Areas (WEAs) and black outlined cross hatched regions are prospective or currently leased OWE call areas, or unsolicited lease areas. Basemap reprinted from World Ocean base under a CC BY license, with permission from Esri, original copyright © 2024. Basemap content is the intellectual property of Esri and is used herein with permission. Copyright © 2024 Esri and its licensors. All rights reserved. S3 Fig. Map series of present value with economic activity (2020 millions USD) of the at-sea hake fishery off the U.S. West Coast. Yellow outlined cross hatched regions are Draft Wind Energy Areas (WEAs) and black outlined cross hatched regions are prospective or currently leased OWE call areas, or unsolicited lease areas. Basemap reprinted from World Ocean base under a CC BY license, with permission from Esri, original copyright © 2024. Basemap content is the intellectual property of Esri and is used herein with permission. Copyright © 2024 Esri and its licensors. All rights reserved. S4 Fig. Map series of present value with economic activity (2020 millions USD) of the shoreside hake fishery off the U.S. West Coast. Yellow outlined cross hatched regions are Draft Wind Energy Areas (WEAs) and black outlined cross hatched regions are prospective or currently leased OWE call areas, or unsolicited lease areas. Basemap reprinted from World Ocean base under a CC BY license, with permission from Esri, original copyright © 2024. Basemap content is the intellectual property of Esri and is used herein with permission. Copyright © 2024 Esri and its licensors. All rights reserved. S5 Fig. Map series of present value with economic activity (2020 millions USD) of the California market squid fishery off the U.S. West Coast. Yellow outlined cross hatched regions are Draft Wind Energy Areas (WEAs) and black outlined cross hatched regions are prospective or currently leased OWE call areas, or unsolicited lease areas. Basemap reprinted from World Ocean base under a CC BY license, with permission from Esri, original copyright © 2024. Basemap content is the intellectual property of Esri and is used herein with permission. Copyright © 2024 Esri and its licensors. All rights reserved. S6 Fig. Map series of present value with economic activity (2020 millions USD) of the pink shrimp fishery off the U.S. West Coast. Yellow outlined cross hatched regions are Draft Wind Energy Areas (WEAs) and black outlined cross hatched regions are prospective or currently leased OWE call areas, or unsolicited lease areas. Basemap reprinted from World Ocean base under a CC BY license, with permission from Esri, original copyright © 2024. Basemap content is the intellectual property of Esri and is used herein with permission. Copyright © 2024 Esri and its licensors. All rights reserved. S7 Fig. Map series of present value with economic activity (2020 millions USD) of the albacore fishery off the U.S. West Coast. Yellow outlined cross hatched regions are Draft Wind Energy Areas (WEAs) and black outlined cross hatched regions are prospective or currently leased OWE call areas, or unsolicited lease areas. Basemap reprinted from World Ocean base under a CC BY license, with permission from Esri, original copyright © 2024. Basemap content is the intellectual property of Esri and is used herein with permission. Copyright © 2024 Esri and its licensors. All rights reserved. S8 Fig. Map series of present value with economic activity (2020 millions USD) of the Chinook fishery off the U.S. West Coast. Yellow outlined cross hatched regions are Draft Wind Energy Areas (WEAs) and black outlined cross hatched regions are prospective or currently leased OWE call areas, or unsolicited lease areas. Basemap reprinted from World Ocean base under a CC BY license, with permission from Esri, original copyright © 2024. Basemap content is the intellectual property of Esri and is used herein with permission. Copyright © 2024 Esri and its licensors. All rights reserved. S9 Fig. Map series of present value with economic activity (2020 millions USD) of the sablefish fishery off the U.S. West Coast. Yellow outlined cross hatched regions are Draft Wind Energy Areas (WEAs) and black outlined cross hatched regions are prospective or currently leased OWE call areas, or unsolicited lease areas. Basemap reprinted from World Ocean base under a CC BY license, with permission from Esri, original copyright © 2024. Basemap content is the intellectual property of Esri and is used herein with permission. Copyright © 2024 Esri and its licensors. All rights reserved. S10 Fig. Map series of present value with economic activity (2020 millions USD) of the California spiny lobster fishery off the U.S. West Coast. Yellow outlined cross hatched regions are Draft Wind Energy Areas (WEAs) and black outlined cross hatched regions are prospective or currently leased OWE call areas, or unsolicited lease areas. Basemap reprinted from World Ocean base under a CC BY license, with permission from Esri, original copyright © 2024. Basemap content is the intellectual property of Esri and is used herein with permission. Copyright © 2024 Esri and its licensors. All rights reserved. S11 Fig. Fishery species’ exposure (mean present value exposed as a percentage of total revenue by fishery, 2020 millions USD) across Pareto optimal sites for different levels of OWE development (Target, gigawatts). 95% confidence intervals are given in corresponding shaded color to species’ trendlines. Targets labeled 2030 and 2045 indicate goals or mandates by individual states (panels (C), (D), and (E)) or regional aggregate goals (panels (A) and (B)). Total revenue by fishery refers to the total revenue at the relevant geographic domain in each panel. Panel (A) represents an unconstrained optimization, where aggregate development targets across all three states are met by siting OWE in waters anywhere within the modeling domain. Panels (C), (D), and (E) represent constrained optimization at the state level for California, Oregon, and Washington, respectively, where their individual targets are met only in state-adjacent waters. Panel (B) represents the sum of panels (C), (D), and (E), where development is summed proportionately based on the relative targets - i.e., 11 GW represents 3 GW each from Washington and Oregon and 5 GW from California. Total fisheries revenue in panel (B) is total regional revenue as in panel (A). S1 Table. Parameters used for InVEST Offshore Wind Energy Production (OWEP) model. S2 Table. Values used for the global wind energy parameters in the InVEST Offshore Wind Energy Production (OWEP) model. S3 Table. Values used in the turbine type parameters for the InVEST Offshore Wind Energy Production (OWEP) model. Turbine parameters were based mainly on a Vestas V236-15.0, 15MW turbine mounted on a floating foundation. Since turbine parameters are not readily available for many turbine types, especially those not currently in mass production, we sourced additional parameters from a 15MW bewind BW 14.xM225 turbine as well as a hub height estimate from an NREL report. S4 Table. Summary table of nine fishery sectors that were represented in the analyses, ranked by proportion of revenue contributed to the U.S. West Coast commercial fisheries industry. Ports marked with *  are essentially U.S. West Coast wide, since the vast majority of landings in the corresponding sectors occur in the states listed. CDFW: California Department of Fish & Wildlife; ODFW: Oregon Department of Fish & Wildlife; WDFW: Washington Department of Fish & Wildlife. S5 Table. Expansion values used for each fishery species and year. S6 Table. Multipliers used for calculating the economic impact (both vessel and processor) of each targeted species, by state and gear type (where applicable).S7 Table. Spearman’s rank correlation rho values and associated statistical significance for each fishery compared with OWE LCOE on a grid cell comparative basis. Negative rho values indicate positive correlation between higher fisheries present value (PV) and presumably more profitable lower energy production cost (lower LCOE). Alternative hypothesis: true rho is not equal to 0.
(DOCX)
