# Supplementary material for: Mapping the value of commercial fishing and potential costs of offshore wind energy on the U.S: West Coast: Towards an assessment of resource use tradeoffs
Source: PLoS One. 2025 Mar 6;20(3):e0315319. doi: 10.1371/journal.pone.0315319 (PMC11884673; doi:10.1371/journal.pone.0315319)
Supplement: S1 File — S1 Fig. Map series of present value with economic activity (2020 millions USD) of the combined fisheries studied off the U.S. West Coast. Yellow outlined cross hatched regions are Draft Wind Energy Areas (WEAs) and black outlined cross hatched regions are prospective or currently leased OWE call areas, or unsolicited lease areas. Basemap reprinted from World Ocean base under a CC BY license, with permission from Esri, original copyright © 2024. Basemap content is the intellectual property of Esri and is used herein with permission. Copyright © 2024 Esri and its licensors. All rights reserved. S2 Fig. Map series of present value with economic activity (2020 millions USD) of the Dungeness crab fishery off the U.S. West Coast. Yellow outlined cross hatched regions are Draft Wind Energy Areas (WEAs) and black outlined cross hatched regions are prospective or currently leased OWE call areas, or unsolicited lease areas. Basemap reprinted from World Ocean base under a CC BY license, with permission from Esri, original copyright © 2024. Basemap content is the intellectual property of Esri and is used herein with permission. Copyright © 2024 Esri and its licensors. All rights reserved. S3 Fig. Map series of present value with economic activity (2020 millions USD) of the at-sea hake fishery off the U.S. West Coast. Yellow outlined cross hatched regions are Draft Wind Energy Areas (WEAs) and black outlined cross hatched regions are prospective or currently leased OWE call areas, or unsolicited lease areas. Basemap reprinted from World Ocean base under a CC BY license, with permission from Esri, original copyright © 2024. Basemap content is the intellectual property of Esri and is used herein with permission. Copyright © 2024 Esri and its licensors. All rights reserved. S4 Fig. Map series of present value with economic activity (2020 millions USD) of the shoreside hake fishery off the U.S. West Coast. Yellow outlined cross hatched regions are Draft Wind Energy Areas (WEAs) and [file pone.0315319.s001.docx]

**Supporting Information**

**Supplementary Results**

### Commercial Fishing

*Dungeness Crab*

The total present value (PV) of Dungeness economic activity was $10.792 B, and value per km^2^ was $230,551 (Table 1). The vast majority of Dungeness crab fishery revenue comes from the region from Monterey, California north through Washington (S2 Fig). Fishing activity was generally concentrated in coastal waters shallower than 150 m (Table 1). Of all the fisheries we analyzed, the Dungeness crab fishery was ranked second in terms of the number of participating vessels. About 1,600 different vessels fished for Dungeness crab from 2011 - 2020 (Table 1), and value maps were based on data from about 75% of those vessels.

*Pacific Oyster*

Since all oyster culture occurs within bays and estuaries, and at depths shallower than 5 m [1,2], overlap with potential OWE was presumed to be not significant or zero.

*Hake*

The total PV of at-sea and shoreside hake economic activity was $5.157 B and $3.137 B, respectively (Table 1). The value per km^2^ was $105,250 for at-sea hake and $60,600 for shoreside hake (Table 1). The vast amount of hake fishing revenue is captured off Washington/Oregon with little to no fishing off the coast of California (S3 and S4 Figs). These two sectors generally operate in deeper water and are constrained by the 1,300 m depth isobath (Table 1). There were far fewer vessels targeting hake, compared with the other species examined (Table 1). From 2011 - 2020, about 200 boats fished for hake, across the at-sea and shoreside sectors. Value mapping was based on data from about 84% of those boats.

*California Market Squid*

The total PV of market squid economic activity was $2.738 B, and value per km^2^ was the highest of all the species studied at $268,011 (Table 1). The vast majority of squid fishing revenue is extracted from the region south of San Francisco, California (S5 Fig). While we were not able to map where fishing for market squid occurred off the coasts of Oregon and Washington, only about 2.5% of the U.S West Coast market squid catch is landed in Oregon and market squid is not fished for or landed off the coast of Washington. This species has the highest value per unit area of all the species and operates almost exclusively in depths shallower than 100 m (Table 1). Market squid was fished by a similar number of boats as hake, at about 240 (Table 1). The fishing value maps were based on data reported by about 70% of those vessels.

*Geoduck*

Since all geoduck landed on the U.S. West Coast are harvested by divers within Puget Sound, Washington, [3,4], revenue from this species in the study area was presumed to be zero.

*Pink Shrimp*

The total PV of this species' economic activity was $3.203 B, and value per km^2^ was $168,238 (Table 1). Revenue from the pink shrimp fishery was highly concentrated off Washington/Oregon, dipping slightly into northern California, and the vessels fish within a narrow depth range of 100-200 m (Table 1, S6 Fig). We were unable to include spatially explicit logbook data for pink shrimp that is landed at California ports, so we were not able to map that activity. Based on the port level landings data, only about 8% of pink shrimp caught off the U.S. West Coast is landed in California ports. Pink shrimp had a smaller number of vessels targeting that species, with about 165 boats (Table 1), and the value maps were based on data from nearly 100% of these vessels.

*Albacore Tuna*

The total PV of albacore economic activity was $2.132 B, and value per km^2^ was the lowest of all the species studied at $4,193 (Table 1). The vast majority of albacore fishing revenue is extracted off Washington/Oregon and northern California, extending all the way out to the EEZ off Washington/Oregon and northern California (S7 Fig). Fishing for this species had the largest footprint of all species analyzed and also occurred extensively at depths greater than 1,300 m (Table 1, S7 Fig). Albacore were ranked third in terms of numbers, with about 1,100 different vessels (Table 1) landing catch for that species between 2011 - 2020. Value mapping was based on data provided by about 95% of those vessels.

*Chinook Salmon*

The total PV of ocean caught Chinook economic activity was $1.126 B, and value per km^2^ was $22,188 (Table 1). Revenue associated with the Chinook salmon fishery is mostly extracted from the coasts of Oregon and central California, and generally occurs in water depths shallower than 200m. The coarse spatial grain of the mapping makes detailed spatial comparisons challenging (S8 Fig). The Chinook salmon fishery had the greatest numbers of vessels targeting that species, at 1,885 (Table 1). Since the value maps for this species were based on data from fish tickets, 100% of the vessels associated with that fishery were represented.

*Sablefish*

The total PV of ocean caught sablefish economic activity was $1.852 B, and value per km^2^ was $20,577 (Table 1). Sablefish revenue was distributed extensively off the West Coast from Cape Mendocino north through Washington (S9 Fig). Similar to the hake fishery, the sablefish fleets generally operate at depths between 200 - 1,300 m (Table 1). Sablefish was the fourth ranked fishery in terms of the number of vessels that target that species. Of the approximately 1,000 boats (Table 1) that landed sablefish during the study time period, value maps were based on data from only about 40% of those vessels.

*California Spiny Lobster*

The total PV of spiny lobster economic activity was $0.960 B, and value per km^2^ was $13,061 (Table 1). The vast majority of spiny lobster fishing revenue was extracted from the area south of Monterey Bay and the fishery generally operates in shallower water (S10 Fig). The species is not fished for or landed at Oregon and Washington ports. The coarser spatial grain of the data makes detailed spatial comparisons challenging. Vessels targeting spiny lobster were at a moderate number, at around 365 (Table 1). The value mapping was based on data provided by about 94% of those vessels.

**References**

1. FAO. *Magallana gigas* [Internet]. Cultured Aquatic Species Information Programme, Fisheries and Aquaculture Division [online]. 2006 [cited 2023 May 14]. Available from: https://www.fao.org/fishery/en/culturedspecies/crassostrea_gigas_

2. Kornbluth A, Perog BD, Crippen S, Zacherl D, Quintana B, Grosholz ED, et al. Mapping oysters on the Pacific coast of North America: A coast-wide collaboration to inform enhanced conservation. PLOS ONE. 2022;17(3):e0263998.

3. WDFW. Commercial wild stock geoduck clam fishery [Internet]. Washington Department of Fish & Wildlife - Fishing and Shellfish - Commercial Fishing. 2023 [cited 2023 Jan 23]. Available from: https://wdfw.wa.gov/fishing/commercial/geoduck

4. WDFW. Geoduck clam (Panopea generosa) [Internet]. Washington Department of Fish & Wildlife - Species & Habitats - Species in Washington. 2023 [cited 2023 Jan 23]. Available from: https://wdfw.wa.gov/species-habitats/species/panopea-generosa

5. Draxl C, Clifton A, Hodge BM, McCaa J. The Wind Integration National Dataset (WIND) Toolkit. Applied Energy. 2015;151:355–66.

6. NOAA. ETOPO2, Global 2 Arc-minute Ocean Depth and Land Elevation from the US National Geophysical Data Center (NGDC) [Internet]. Boulder, CO USA: Research Data Archive at the National Center for Atmospheric Research, Computational and Information Systems Laboratory; 2001 [cited 2022 May 4]. Available from: https://doi.org/10.5065/D6668B75

7. Stehly T, Duffy P. 2021 Cost of Wind Energy Review [Internet]. National Renewable Energy Laboratory; 2022 Dec p. 64. Available from: https://www.nrel.gov/docs/fy23osti/84774.pdf

8. USDOE. Offshore Wind Market Report: 2022 Edition [Internet]. U.S. Department of Energy, Office of Energy Efficiency & Renewable Energy; 2022 [cited 2023 Feb 10] p. 110. Available from: https://www.energy.gov/sites/default/files/2022-09/offshore-wind-market-report-2022-v2.pdf

**Supplementary Tables**

**S1 Table: Parameters used for InVEST Offshore Wind Energy Production (OWEP) model.**

| **Parameter** | **Value or file** | **Source** |
| --- | --- | --- |
| Wind Data Points | NREL_HourlyWind_Pacific_2017_01_22.csv, provides shape and scale parameters of estimated Weibull distribution for wind speeds across Pacific coast BOEM aliquots (1200m^2^) for the period 2007 - 2013 [5]. | NREL WINDKIT <https://cscdata.nrel.gov/#/datasets/3cb55fd7-57ec-418c-a8ba-181e4a2779b2> |
| Area of Interest | srtm30_cclme_aoi_utm_z11.shp | This analysis |
| Bathymetric Digital Elevation Model | global_dem.tif | [6] |
| Land Polygon for Distance Calculation | global_polygon_clipped_geo.shp | This analysis |
| Global Wind Energy Parameters File | global_wind_energy_parameters_re_2020USD.csv (see Table 2 for details) | This analysis, see Table 2 for details |
| Turbine Type Parameters File | 15_0_prototype_turbine.csv (see Table 3 for details) | This analysis, see Table 3 for details |
| Number of Turbines | 60 | This analysis |
| Min Depth for Wind Farm (m) | 60 | BOEM |
| Max Depth for Wind Farm (m) | 1600* | BOEM |
| Min Distance for Wind Farm (m) | 0 | This analysis |
| Max Distance for Wind Farm (km) | 100 | This analysis |
| Cost of Foundation (USD, M) | 31.335 | [7] |
| Discount Rate | 0.052 | [8] |
| Average Shore to Grid Distance (km) | 1 | Default |

* The 1,600 m depth cutoff in the InVEST bathymetry grid better approximates the actual 1,300 m isobath, given the coarse spatial resolution of the bathymetry.

**S2 Table: Values used for the global wind energy parameters in the InVEST Offshore Wind Energy Production (OWEP) model.**

| **Parameter** | **Value** | **Source** |
| --- | --- | --- |
| air_density | 1.225 | Default, see InVEST user’s guide (https://storage.googleapis.com/releases.naturalcapitalproject.org/invest-userguide/latest/en/index.html) for more detail for default parameters |
| exponent_power_curve | 2 | Default |
| decommission_cost | 0.0244 | Based on decommissioning cost represented as a proportion of total CAPEX costs (turbine + foundation + electrical infrastructure) from NREL 2021 Cost of Wind Energy Review <https://www.nrel.gov/docs/fy23osti/84774.pdf> |
| operation_maintenance_cost | 0.035 | Default |
| miscellaneous_capex_cost | 0.0471 | Cost proportion of total CAPEX costs (turbine + foundation + electrical infrastructure) for development, project management, construction insurance, and commissioning from NREL 2021 Cost of Wind Energy Review <https://www.nrel.gov/docs/fy23osti/84774.pdf> |
| installation_cost | 0.0764 | Based on assembly and installation cost as a proportion of total CAPEX costs (turbine + foundation + electrical infrastructure) from NREL 2021 Cost of Wind Energy Review <https://www.nrel.gov/docs/fy23osti/84774.pdf> |
| infield_cable_length | 0.91 | Default |
| infield_cable_cost | 0.25 | Default, adjusted to 2020 USD using producer price index (https://fred.stlouisfed.org/series/PPIACO#0) |
| mw_coef_ac | 0.7788 | Default, adjusted to 2020 USD using producer price index (https://fred.stlouisfed.org/series/PPIACO#0) |
| mw_coef_dc | 1.048 | Default, adjusted to 2020 USD using producer price index (https://fred.stlouisfed.org/series/PPIACO#0) |
| cable_coef_ac | 1.3076 | Default, adjusted to 2020 USD using producer price index (https://fred.stlouisfed.org/series/PPIACO#0) |
| cable_coef_dc | 0.8557 | Default, adjusted to 2020 USD using producer price index (https://fred.stlouisfed.org/series/PPIACO#0) |
| ac_dc_distance_break | 60 | Default |
| time_period | 33 | Most common length operating period quoted on recent BOEM lease sales |
| rotor_diameter_factor | 7 | Default |
| carbon_coefficient | 0.00068956 | Default |
| air_density_coefficient | 0.0001194 | Default |
| loss_parameter | 0.05 | Default |

**S3 Table: Values used in the turbine type parameters for the InVEST Offshore Wind Energy Production (OWEP) model.** Turbine parameters were based mainly on a Vestas V236-15.0, 15MW turbine mounted on a floating foundation. Since turbine parameters are not readily available for many turbine types, especially those not currently in mass production, we sourced additional parameters from a 15MW bewind BW 14.xM225 turbine as well as a hub height estimate from an NREL report.

| **Parameter** | **Value** | **Source** |
| --- | --- | --- |
| Hub height (m) | 160 | <https://www.nrel.gov/docs/fy22osti/83434.pdf> |
| Cut in wind speed (m/s) | 3 | <https://en.wind-turbine-models.com/turbines/2317-vestas-v236-15.0> |
| Rated wind speed (m/s) | 12 | <https://en.wind-turbine-models.com/turbines/2288-bewind-bw-14.xm225> |
| Cut out wind speed (m/s) | 30 | <https://en.wind-turbine-models.com/turbines/2317-vestas-v236-15.0> |
| Rated power (MW) | 15 | <https://en.wind-turbine-models.com/turbines/2317-vestas-v236-15.0> |
| Cost (M USD) | 19.52 | <https://en.wind-turbine-models.com/turbines/2317-vestas-v236-15.0> |
| Turbines per circuit | 6 | Default |
| Rotor diameter (m) | 236 | <https://en.wind-turbine-models.com/turbines/2317-vestas-v236-15.0> |

**S4 Table: Summary table of nine fishery sectors that were represented in the analyses, ranked by proportion of revenue contributed to the U.S. West Coast commercial fisheries industry.** Ports marked with * are essentially U.S. West Coast wide, since the vast majority of landings in the corresponding sectors occur in the states listed. CDFW: California Department of Fish & Wildlife; ODFW: Oregon Department of Fish & Wildlife; WDFW: Washington Department of Fish & Wildlife.

| **Fishery species** | **Ports Represented** | **Data Type** | **Source Type** | **Source(s) of data** |
| --- | --- | --- | --- | --- |
| **Dungeness**  **crab** (pot) | Washington | Logbook | State | WDFW, PacFIN |
|  | Oregon | Logbook | State | ODFW, PacFIN |
|  | California | VMS | Federal | CDFW, OLE, PacFIN |
| **Hake**, at-sea (mid-water trawl) | West Coast | Observer | Federal | NWFSC Observer Program, PacFIN |
| **Hake**, shoreside (mid-water trawl) | West Coast | Observer,  Electronic Monitoring | Federal | NWFSC Observer Program, electronic monitoring, PacFIN |
| **California market squid** (seine) | *California | Logbook | State | CDFW logbook, PacFIN |
| **Pink shrimp** (shrimp trawl) | *Washington | Logbook | State | WDFW logbook, PacFIN |
|  | *Oregon | Logbook | State | ODFW logbook, PacFIN |
| **Albacore** (troll/hook-and-line) | West Coast | Logbook | Federal | NMFS SWFSC, PacFIN |
| **Chinook salmon** (troll) | West Coast | Blue Book | PFMC | PFMC, PacFIN |
| **Sablefish** (trawl & fixed gear - pot) | West Coast | Observer,  Electronic Monitoring | Federal | NWFSC Observer Program, electronic monitoring, PacFIN |
| **California spiny lobster** (pot) | *California | Fish tickets | State | CDFW logbook, PacFIN |

**S5 Table: Expansion values used for each fishery species and year.**

**
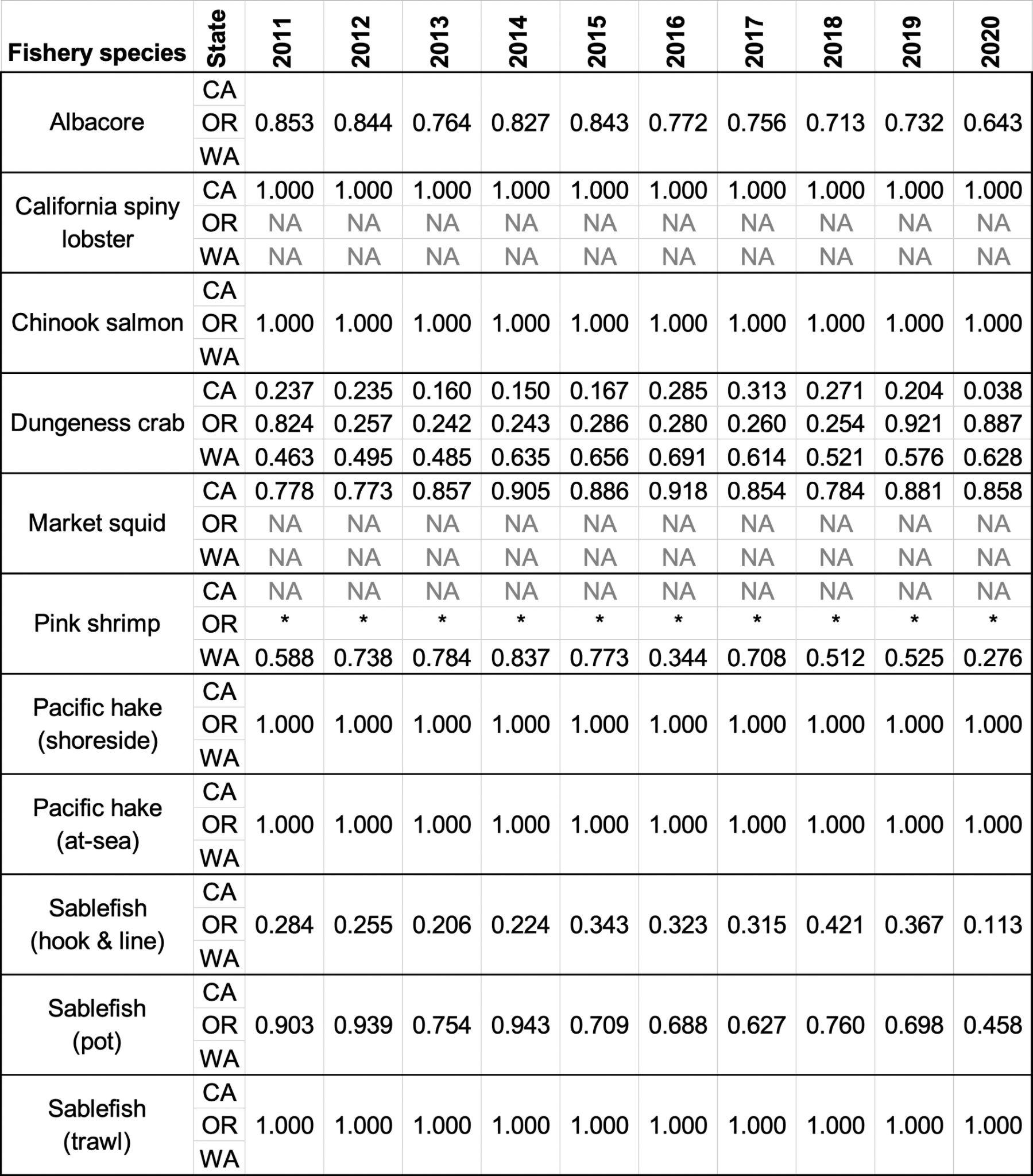
**

* indicates expansion corrections provided by the data source agency.

**S6 Table: Multipliers used for calculating the economic impact (both vessel and processor) of each targeted species, by state and gear type (where applicable).**


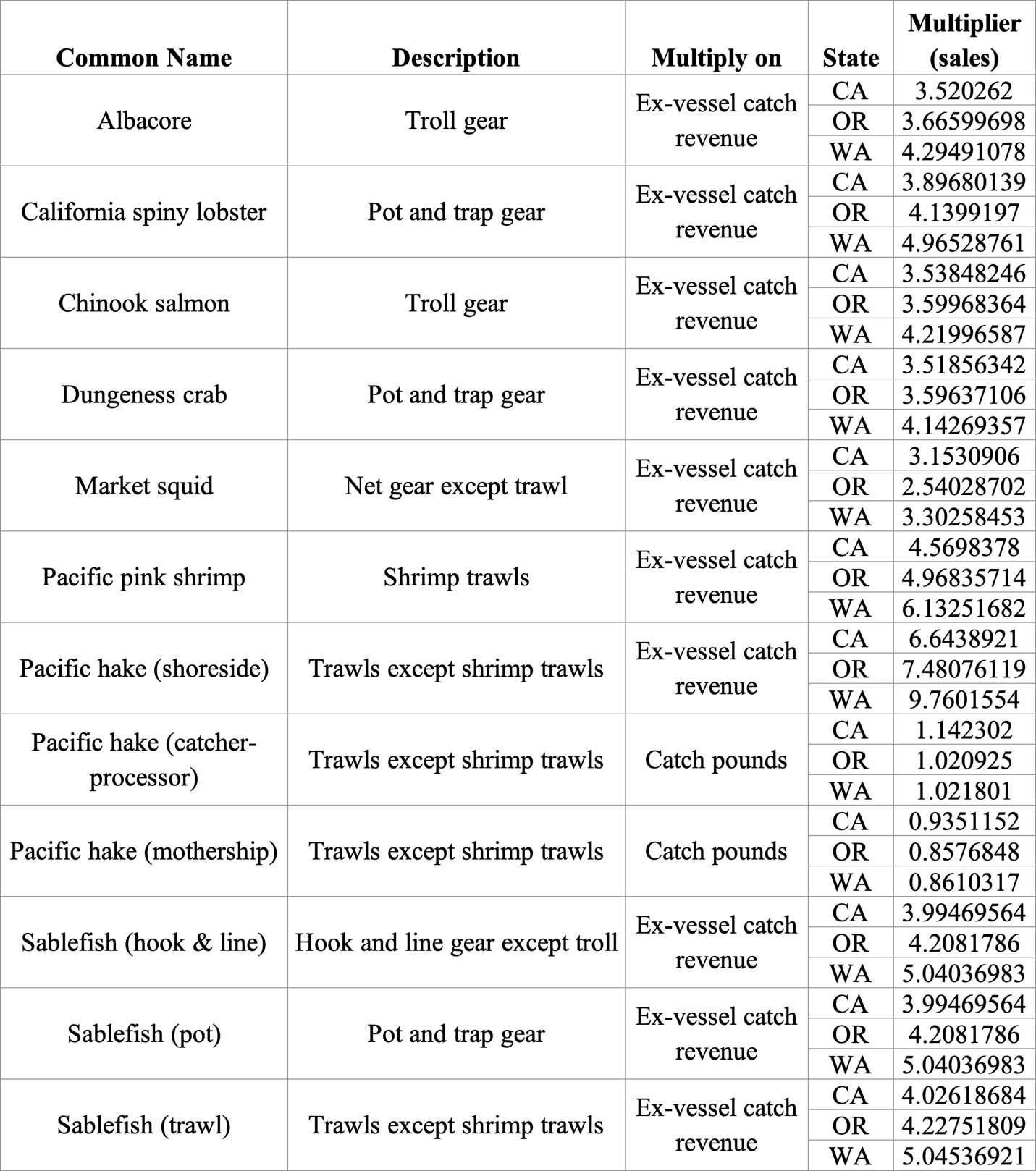


**S7 Table: Spearman's rank correlation rho values and associated statistical significance for each fishery compared with OWE LCOE on a grid cell comparative basis.** Negative rho values indicate positive correlation between higher fisheries present value (PV) and presumably more profitable lower energy production cost (lower LCOE). Alternative hypothesis: true rho is not equal to 0.

| **Fishery** | **rho** | **p-value** | **S** |
| --- | --- | --- | --- |
| All species | -0.160334 | < 2.2E-16 | 1.0204E+12 |
| Dungeness crab | 0.293356 | < 2.2E-16 | 1.0094E+10 |
| Hake (at-sea) | -0.259565 | < 2.2E-16 | 1.2443E+10 |
| Hake (shoreside) | 0.210842 | < 2.2E-16 | 2.8509E+10 |
| Market squid | 0.144284 | 5.424E-05 | 6.6902E+07 |
| Pink shrimp | -0.071932 | 5.942E-04 | 2.1064E+09 |
| Albacore | -0.091648 | < 2.2E-16 | 2.0768E+11 |
| Chinook salmon | 0.125119 | < 2.2E-16 | 1.6106E+10 |
| Sablefish | -0.196540 | < 2.2E-16 | 2.1602E+11 |
| Spiny lobster | 0.433301 | < 2.2E-16 | 2.0698E+10 |

**Supplementary Figures**


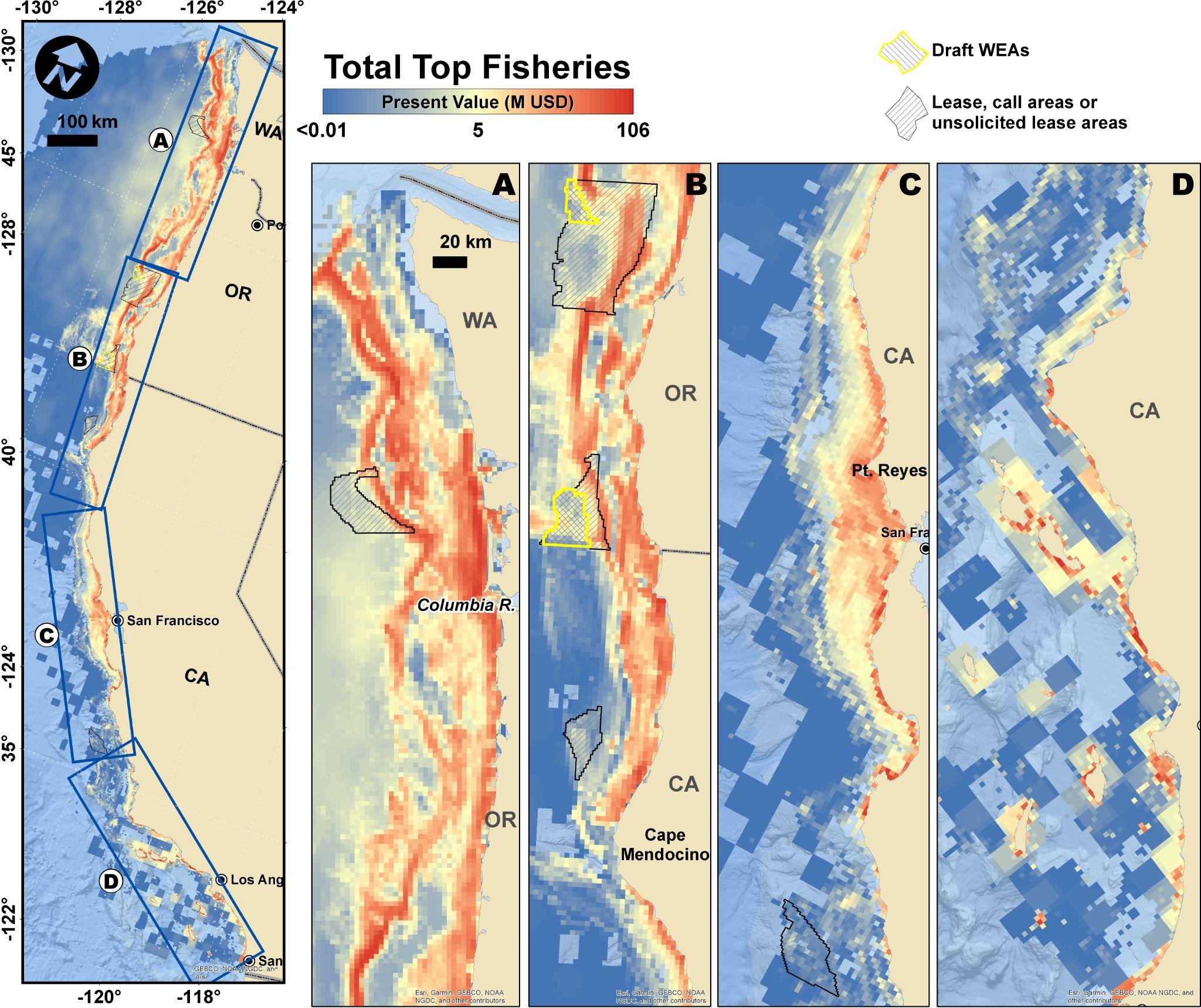


**S1 Fig. Map series of present value with economic activity (2020 millions USD) of the combined fisheries studied off the U.S. West Coast.** Yellow outlined cross hatched regions are Draft Wind Energy Areas (WEAs) and black outlined cross hatched regions are prospective or currently leased OWE call areas, or unsolicited lease areas. Basemap reprinted from World Ocean base under a CC BY license, with permission from Esri, original copyright © 2024. Basemap content is the intellectual property of Esri and is used herein with permission. Copyright © 2024 Esri and its licensors. All rights reserved.


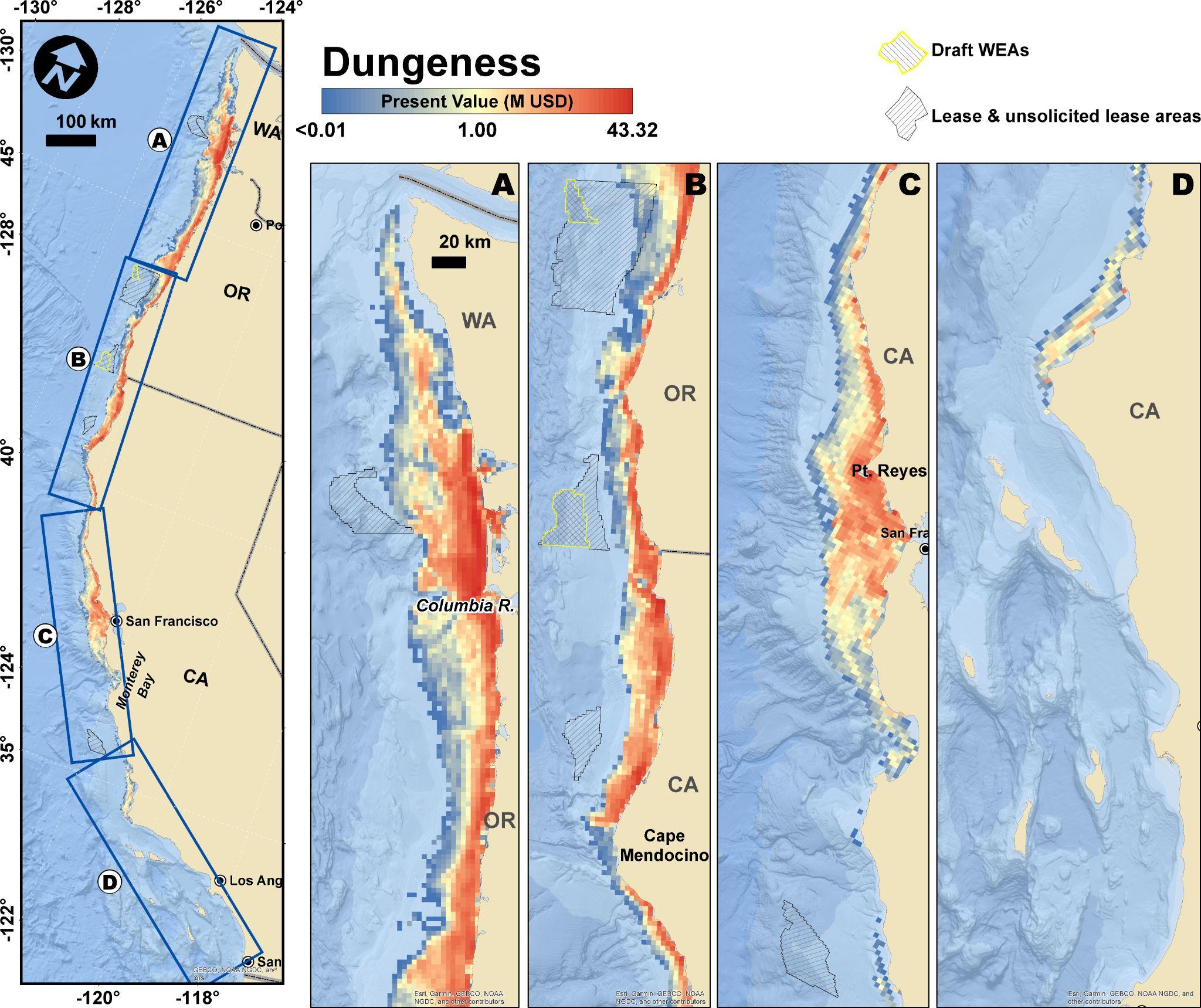


**S2 Fig. Map series of present value with economic activity (2020 millions USD) of the Dungeness crab fishery off the U.S. West Coast.** Yellow outlined cross hatched regions are Draft Wind Energy Areas (WEAs) and black outlined cross hatched regions are prospective or currently leased OWE call areas, or unsolicited lease areas. Basemap reprinted from World Ocean base under a CC BY license, with permission from Esri, original copyright © 2024. Basemap content is the intellectual property of Esri and is used herein with permission. Copyright © 2024 Esri and its licensors. All rights reserved.


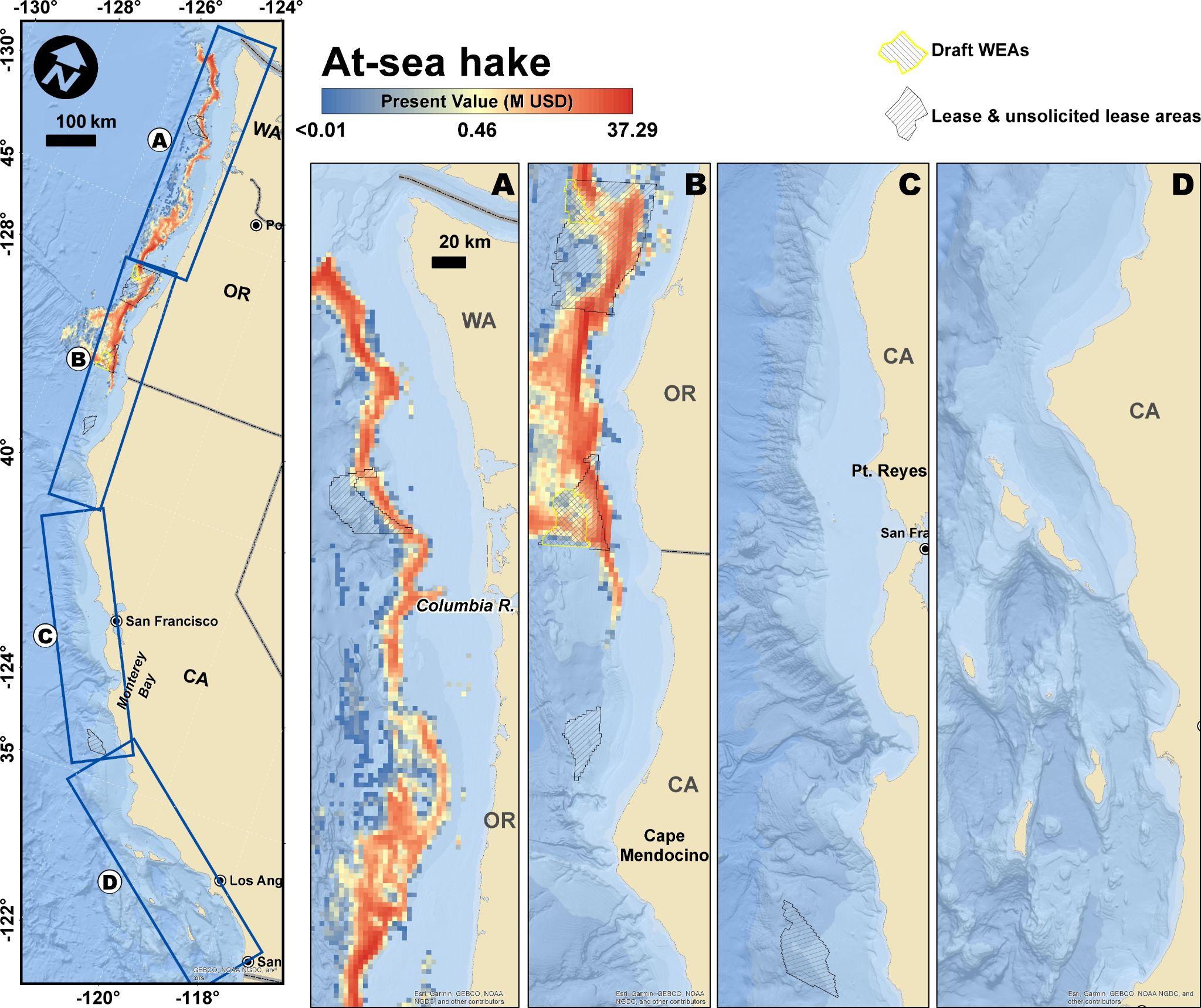


**S3 Fig. Map series of present value with economic activity (2020 millions USD) of the at-sea hake fishery off the U.S. West Coast.** Yellow outlined cross hatched regions are Draft Wind Energy Areas (WEAs) and black outlined cross hatched regions are prospective or currently leased OWE call areas, or unsolicited lease areas. Basemap reprinted from World Ocean base under a CC BY license, with permission from Esri, original copyright © 2024. Basemap content is the intellectual property of Esri and is used herein with permission. Copyright © 2024 Esri and its licensors. All rights reserved.


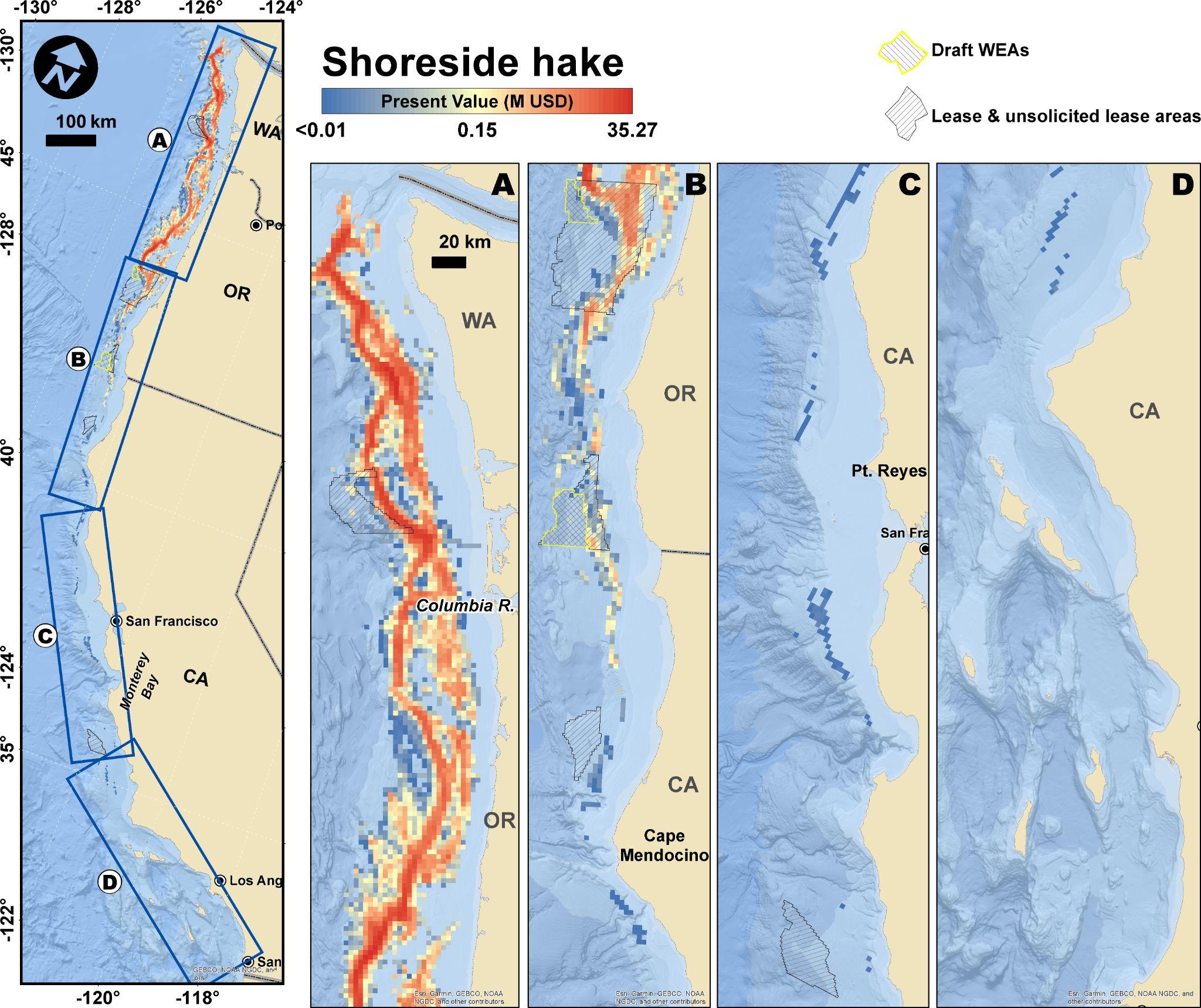


**S4 Fig. Map series of present value with economic activity (2020 millions USD) of the shoreside hake fishery off the U.S. West Coast.** Yellow outlined cross hatched regions are Draft Wind Energy Areas (WEAs) and black outlined cross hatched regions are prospective or currently leased OWE call areas, or unsolicited lease areas. Basemap reprinted from World Ocean base under a CC BY license, with permission from Esri, original copyright © 2024. Basemap content is the intellectual property of Esri and is used herein with permission. Copyright © 2024 Esri and its licensors. All rights reserved.


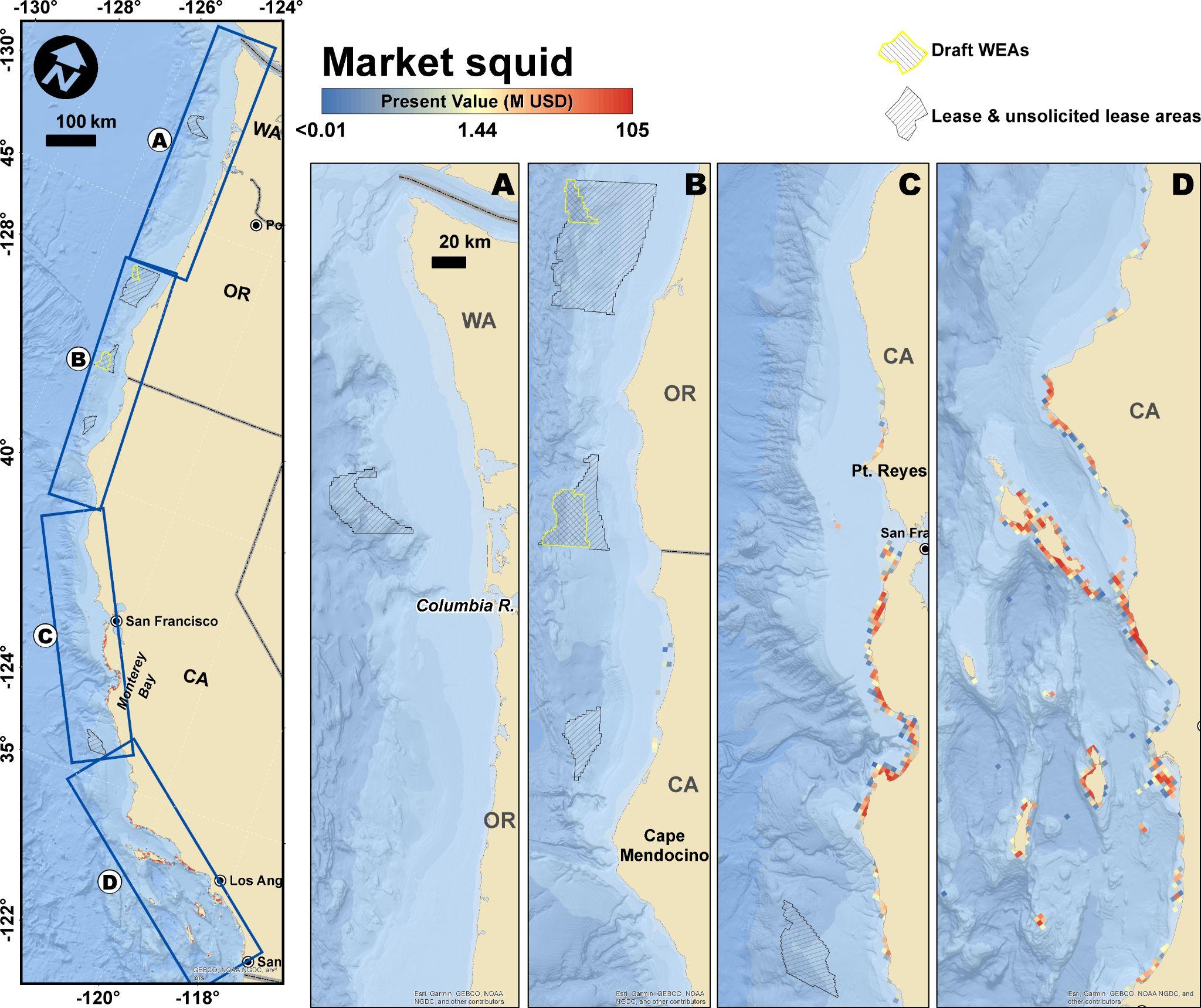


**S5 Fig. Map series of present value with economic activity (2020 millions USD) of the California market squid fishery off the U.S. West Coast.** Yellow outlined cross hatched regions are Draft Wind Energy Areas (WEAs) and black outlined cross hatched regions are prospective or currently leased OWE call areas, or unsolicited lease areas. Basemap reprinted from World Ocean base under a CC BY license, with permission from Esri, original copyright © 2024. Basemap content is the intellectual property of Esri and is used herein with permission. Copyright © 2024 Esri and its licensors. All rights reserved.


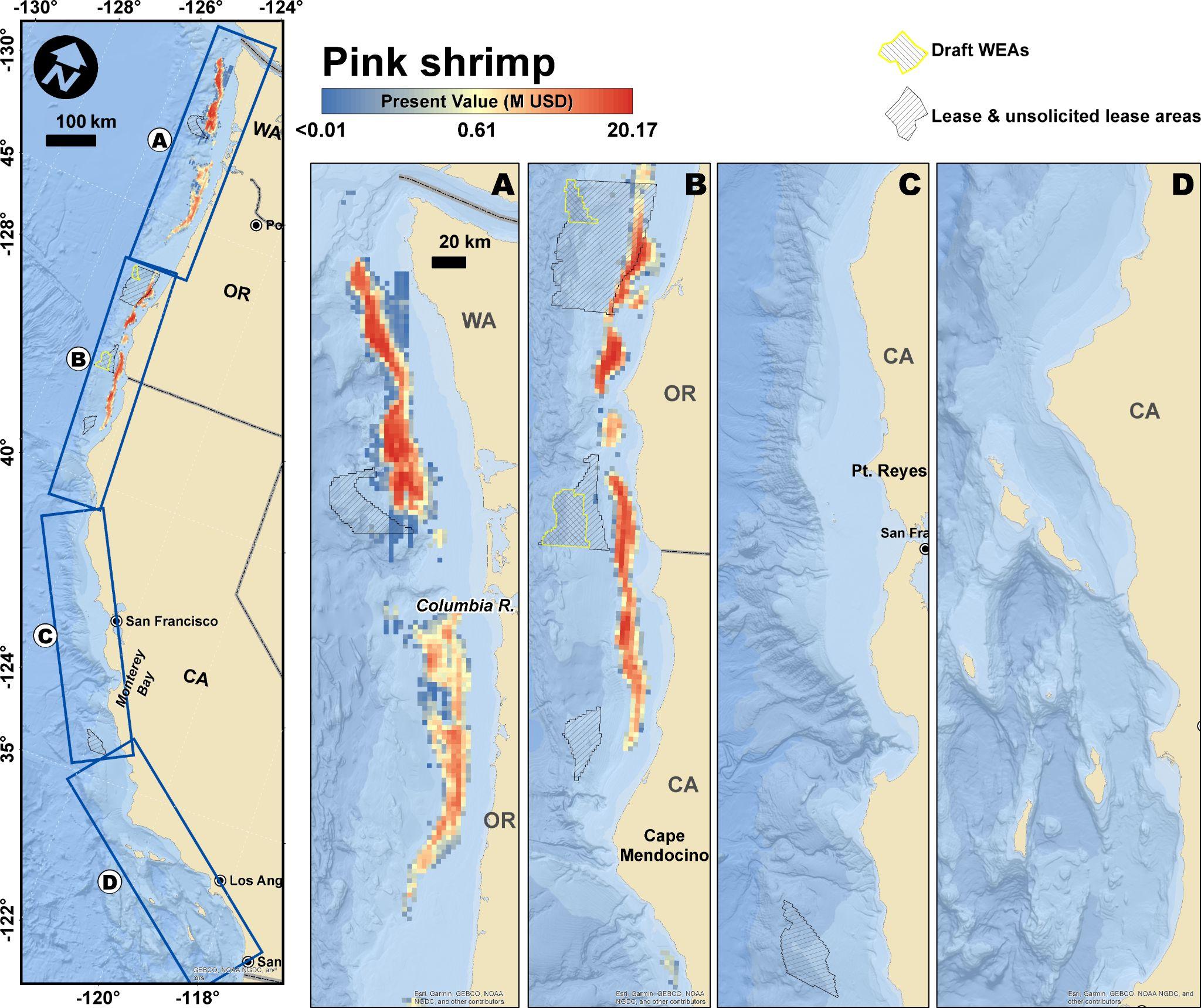


**S6 Fig. Map series of present value with economic activity (2020 millions USD) of the pink shrimp fishery off the U.S. West Coast.** Yellow outlined cross hatched regions are Draft Wind Energy Areas (WEAs) and black outlined cross hatched regions are prospective or currently leased OWE call areas, or unsolicited lease areas. Basemap reprinted from World Ocean base under a CC BY license, with permission from Esri, original copyright © 2024. Basemap content is the intellectual property of Esri and is used herein with permission. Copyright © 2024 Esri and its licensors. All rights reserved.


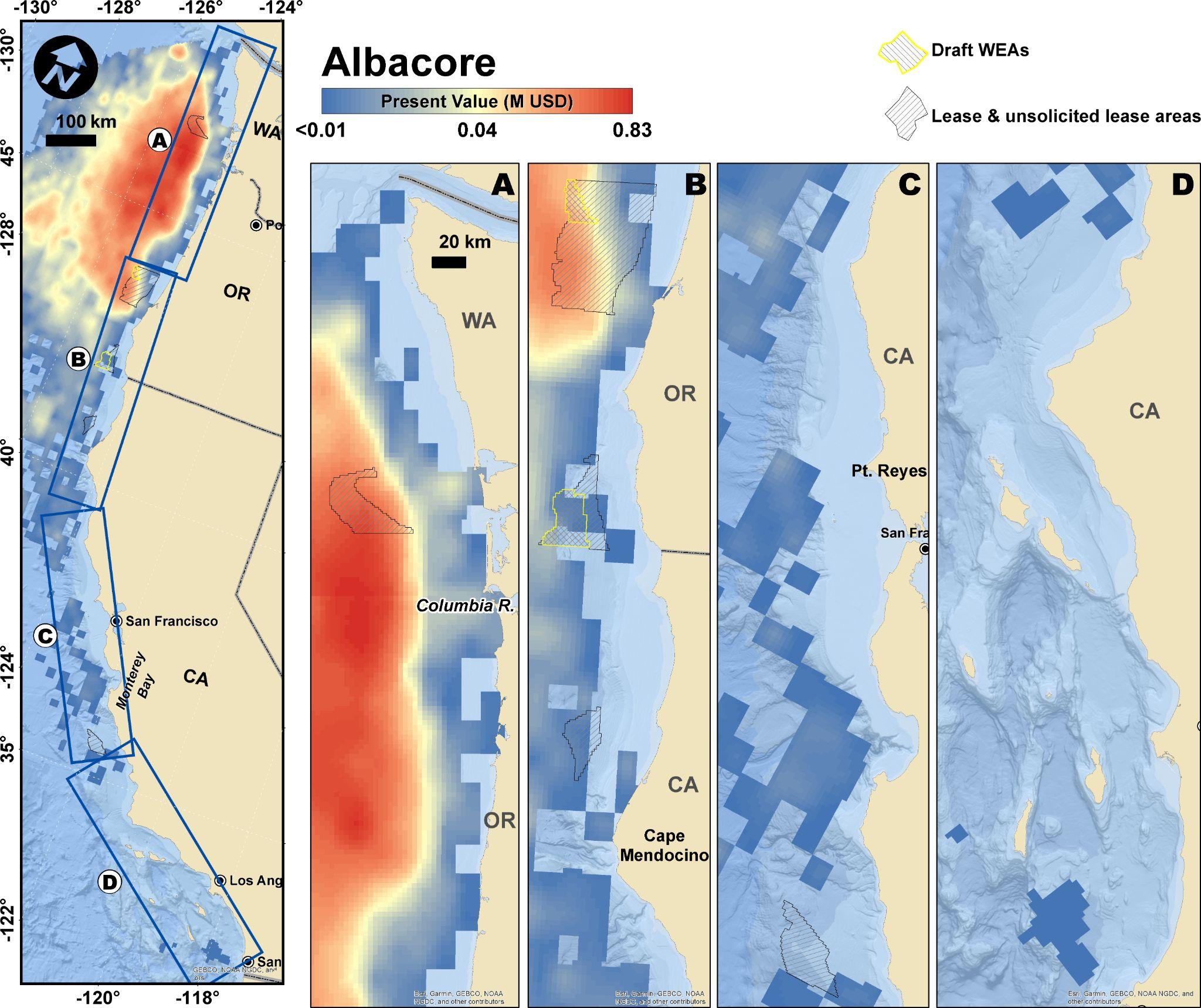


**S7 Fig. Map series of present value with economic activity (2020 millions USD) of the albacore fishery off the U.S. West Coast.** Yellow outlined cross hatched regions are Draft Wind Energy Areas (WEAs) and black outlined cross hatched regions are prospective or currently leased OWE call areas, or unsolicited lease areas. Basemap reprinted from World Ocean base under a CC BY license, with permission from Esri, original copyright © 2024. Basemap content is the intellectual property of Esri and is used herein with permission. Copyright © 2024 Esri and its licensors. All rights reserved.


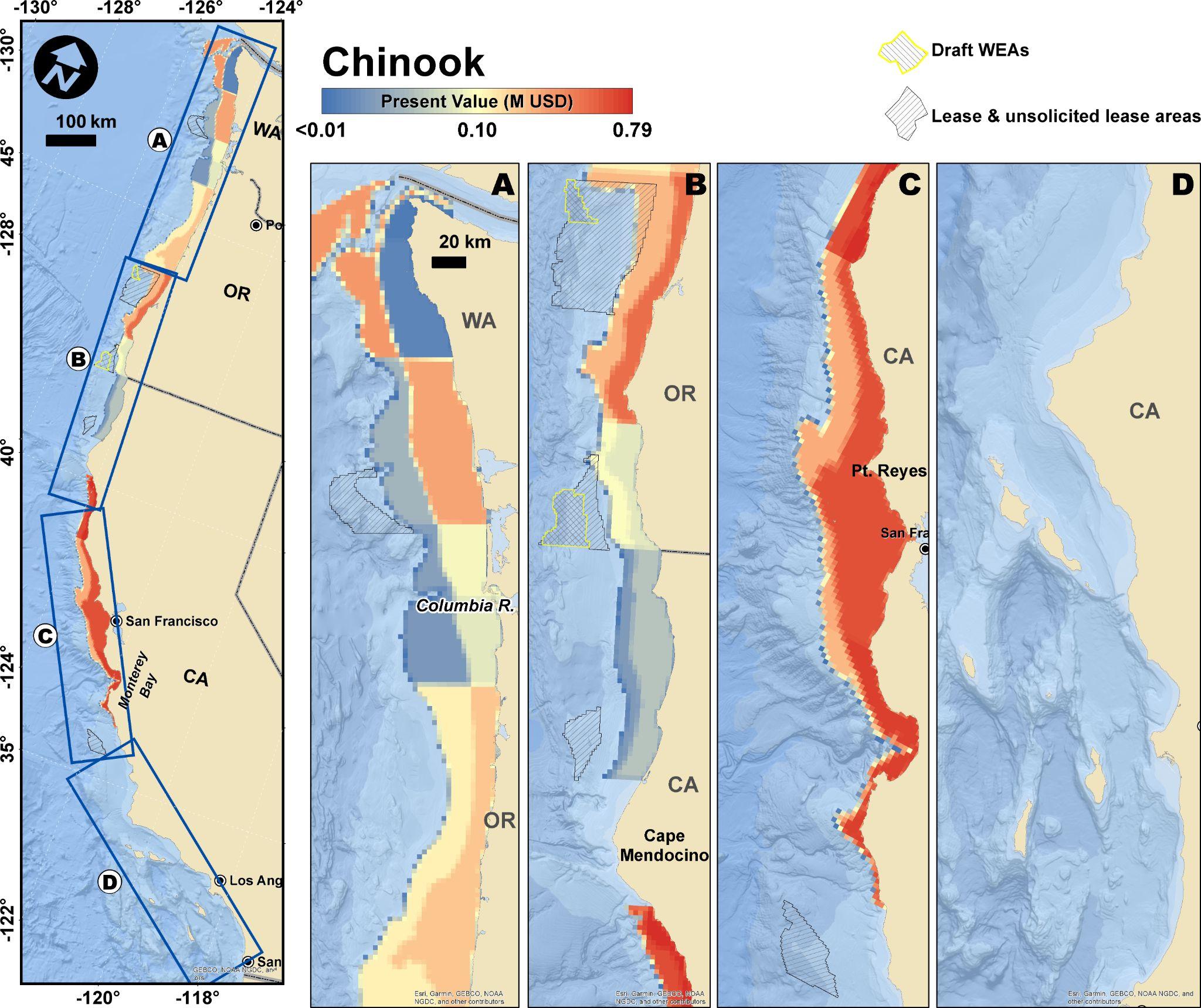


**S8 Fig. Map series of present value with economic activity (2020 millions USD) of the Chinook fishery off the U.S. West Coast.** Yellow outlined cross hatched regions are Draft Wind Energy Areas (WEAs) and black outlined cross hatched regions are prospective or currently leased OWE call areas, or unsolicited lease areas. Basemap reprinted from World Ocean base under a CC BY license, with permission from Esri, original copyright © 2024. Basemap content is the intellectual property of Esri and is used herein with permission. Copyright © 2024 Esri and its licensors. All rights reserved.


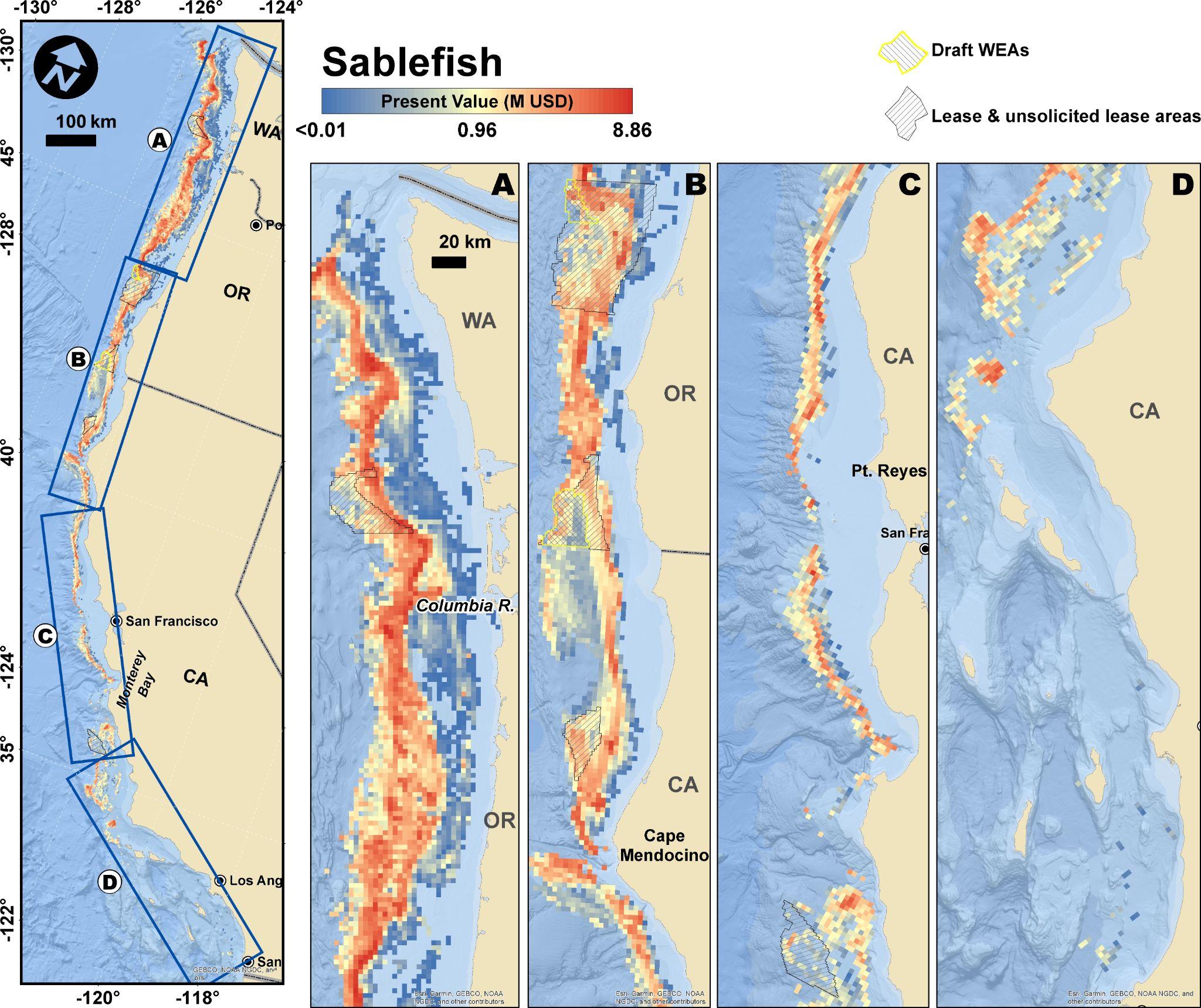


**S9 Fig. Map series of present value with economic activity (2020 millions USD) of the sablefish fishery off the U.S. West Coast.** Yellow outlined cross hatched regions are Draft Wind Energy Areas (WEAs) and black outlined cross hatched regions are prospective or currently leased OWE call areas, or unsolicited lease areas. Basemap reprinted from World Ocean base under a CC BY license, with permission from Esri, original copyright © 2024. Basemap content is the intellectual property of Esri and is used herein with permission. Copyright © 2024 Esri and its licensors. All rights reserved.


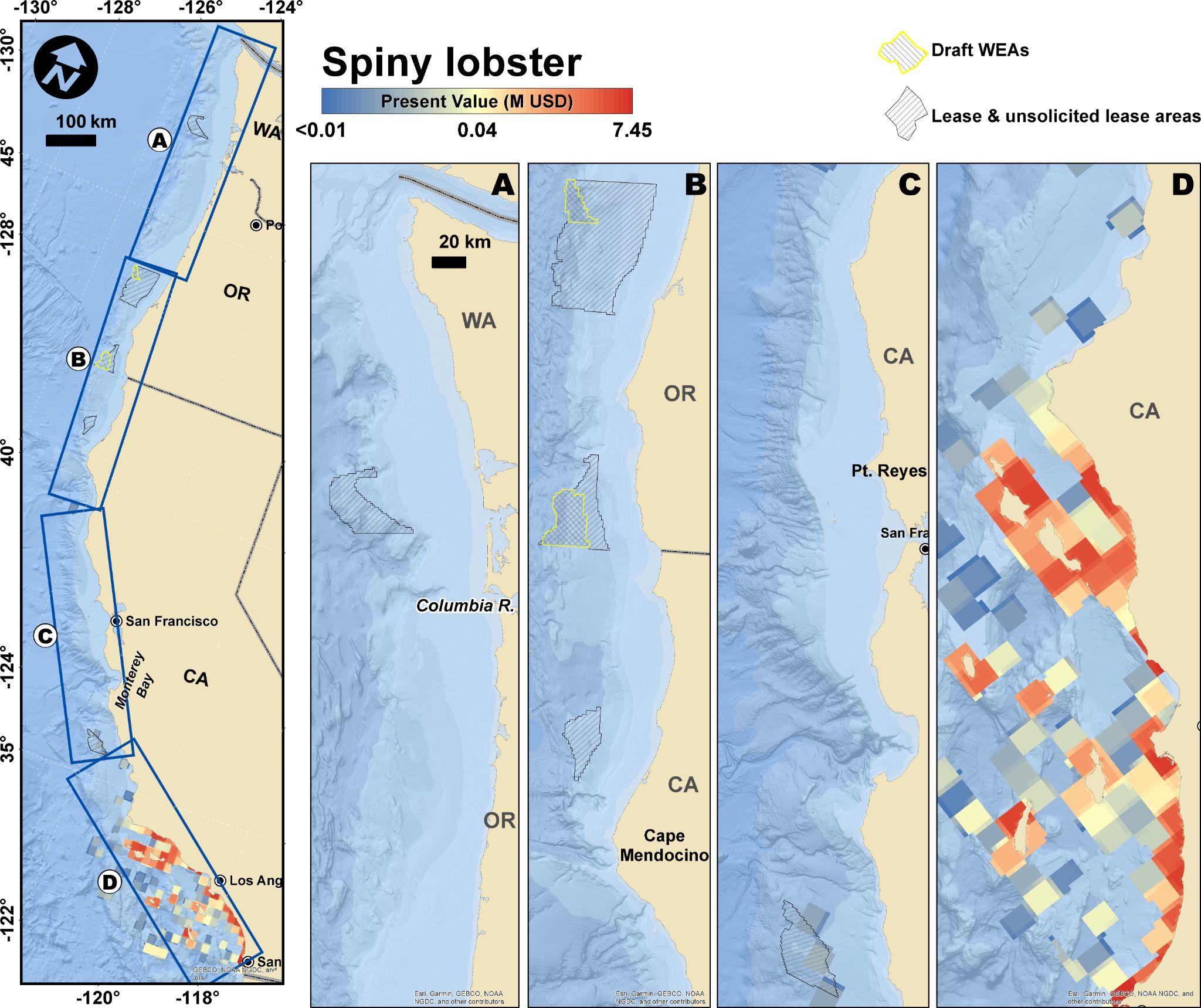


**S10 Fig. Map series of present value with economic activity (2020 millions USD) of the California spiny lobster fishery off the U.S. West Coast.** Yellow outlined cross hatched regions are Draft Wind Energy Areas (WEAs) and black outlined cross hatched regions are prospective or currently leased OWE call areas, or unsolicited lease areas. Basemap reprinted from World Ocean base under a CC BY license, with permission from Esri, original copyright © 2024. Basemap content is the intellectual property of Esri and is used herein with permission. Copyright © 2024 Esri and its licensors. All rights reserved.


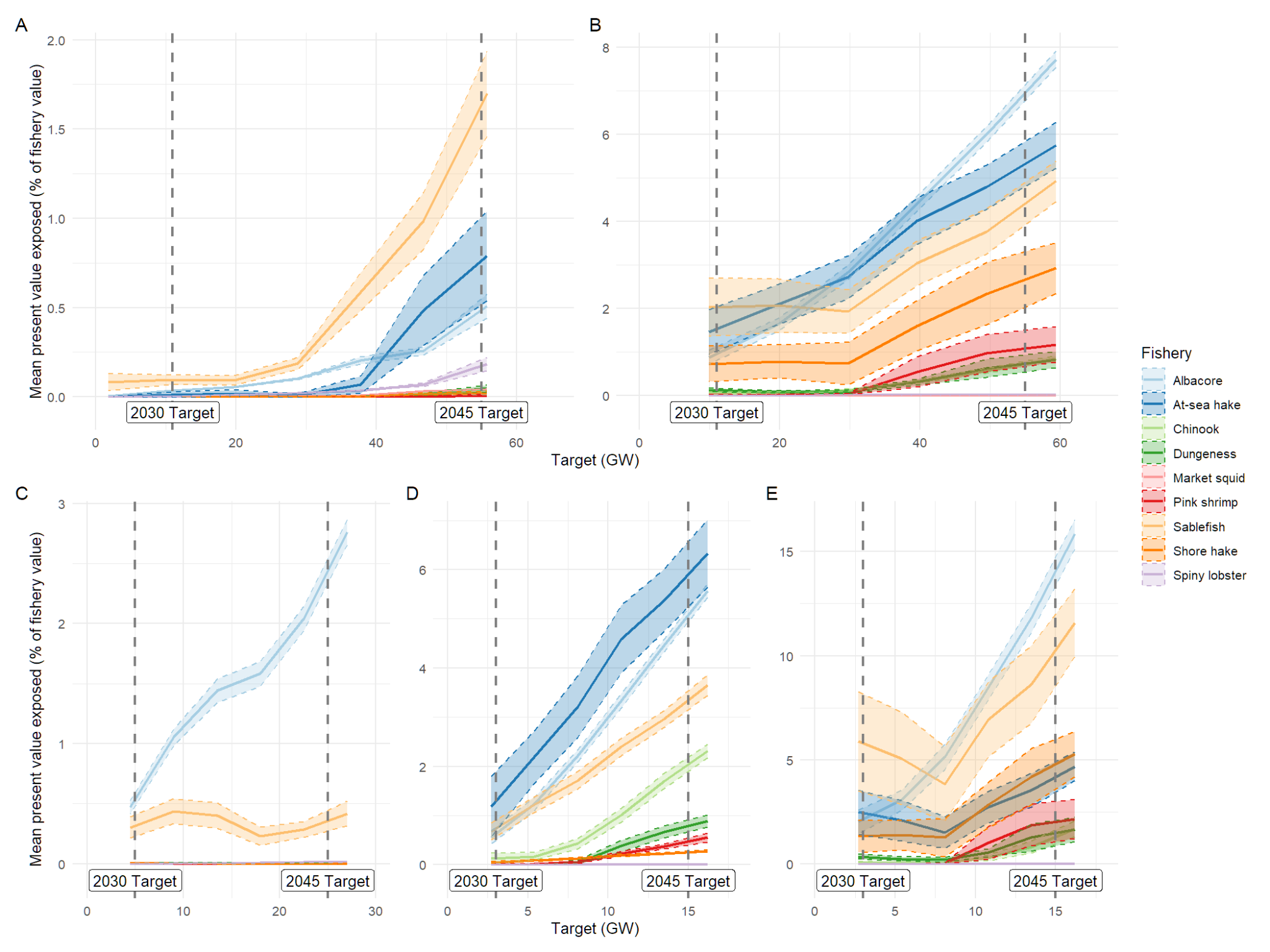


**S11 Fig. Fishery species' exposure (mean present value exposed as a percentage of total revenue by fishery, 2020 millions USD) across Pareto optimal sites for different levels of OWE development (Target, gigawatts).** 95% confidence intervals are given in corresponding shaded color to species’ trendlines. Targets labeled 2030 and 2045 indicate goals or mandates by individual states (panels (C), (D), and (E)) or regional aggregate goals (panels (A) and (B)). Total revenue by fishery refers to the total revenue at the relevant geographic domain in each panel. Panel (A) represents an unconstrained optimization, where aggregate development targets across all three states are met by siting OWE in waters anywhere within the modeling domain. Panels (C), (D), and (E) represent constrained optimization at the state level for California, Oregon, and Washington, respectively, where their individual targets are met only in state-adjacent waters. Panel (B) represents the sum of panels (C), (D), and (E), where development is summed proportionately based on the relative targets - i.e., 11 GW represents 3 GW each from Washington and Oregon and 5 GW from California. Total fisheries revenue in panel (B) is total regional revenue as in panel (A).
